# Inducible CD147 up-regulation boosts extended SARS-CoV-2 infection triggering severe COVID-19 independent of ACE2

**DOI:** 10.1038/s41392-025-02551-x

**Published:** 2026-02-03

**Authors:** Ke Wang, Peng Lin, Ruo Chen, Qiang Huang, Yizhen Zhao, Lei Zhang, Yongxiang Zhao, Liping Zhong, Ke Xu, Linlin Bao, Youchun Wang, Chuan Qin, Guizhen Wu, Hai Zhang, Jiejie Geng, Zheng Zhang, Ding Wei, Xiaochun Chen, Hao Tang, Liu Yang, Xu Yang, Xiuxuan Sun, Rui Yao, Ye Zhao, Weijun Qin, Zhiwei Yang, Liang Chen, Huijie Bian, Zhi-Nan Chen, Ping Zhu

**Affiliations:** 1 Department of Cell Biology of National Translational Science Center for Molecular Medicine and Department of Clinical Immunology of Xijing Hospital, Fourth Military Medical University, Xi’an, China; 2 State Key Laboratory of New Targets Discovery and Drug Development for Major Diseases, Xi’an, China; 3 School of Medicine, Shanghai University, Shanghai, China; 4 MOE Key Laboratory for Nonequilibrium Synthesis and Modulation of Condensed Matter, School of Physics, Xi’an Jiaotong University, Xi’an, China; 5 National Center for International Research of Bio-targeting Theranostics, Guangxi Key Laboratory of Biotargeting Theranostics, Collaborative Innovation Center for Targeting Tumor Diagnosis and Therapy, Guangxi Medical University, Nanning, China; 6 National Institute for Viral Disease Control and Prevention, Chinese Center for Disease Control and Prevention, Beijing, China; 7 Beijing Key Laboratory for Animal Models of Emerging and Remerging Infectious Diseases, NHC Key Laboratory of Human Disease Comparative Medicine, Institute of Laboratory Animal Science, Chinese Academy of Medical Sciences and Comparative Medicine Center, Peking Union Medical College, Beijing, China; 8 Institute of Medical Biology, Chinese Academy of Medical Sciences and Peking Union Medical College, Kunming, China; 9 Jiangsu Pacific Meinuoke Biopharmaceutical Co., Ltd., Changzhou, China; 10 Department of Urology, Xijing Hospital, Fourth Military Medical University, Xi’an, China

**Keywords:** Infection, Inflammation

## Abstract

The high mortality caused by severe COVID-19 poses great challenges to the public health. However, the underlying pathogenesis of severe cases remains unclear. Here, we find that SARS-CoV-2 infection boosts CD147 inducible up-regulation in the lung tissues of virus-infected rhesus macaques coupled with down-regulated membrane-bound ACE2, which conduces to extended virus infection and severe pathological lesions. Specifically, SARS-CoV-2 infection enhances the expression of transcriptional factor aryl hydrocarbon receptor and facilitates its nucleus translocation, which causes CD147 gene transcription and its up-regulation in protein level, thereby leading to virus susceptibility of the hosts and extended virus infection. Meanwhile, SARS-CoV-2 infection triggers immune imbalance of lung tissues by promoting cell death of CD4 + T cells and B cells and mediating abnormal cell-cell communications, especially for M2 macrophages. Meplazumab, a humanized anti-CD147 antibody, effectively inhibits virus entry and cytokine level, and restores immune balance in the lung tissues of virus-infected rhesus macaque model. Importantly, we further present the cryo-EM structure of CD147-spike complex, and identify five pairs of functional residues for their interaction, which could be interrupted by Meplazumab via steric hindrance effect. Our findings provide direct evidence for CD147-SARS-CoV-2 spike interaction and uncover the pathogenesis of severe COVID-19 caused by CD147-mediated extended virus infection.

## Introduction

Severe COVID-19, characterized by high virus loads and immune dysfunction, contributes to high mortality among hospitalized individuals and continues to challenge the public health and economic stability.^1–9^ Although the current COVID-19 pandemic has gradually subsided, severe infections still exist, especially for those with chronic diseases and poor immune conditions. It has been reported that the virus enters host cells through receptor-dependent membrane fusion or endocytosis, and the progeny virions are exocytosed for the next round of infection, thereby leading to continuous virus infection within the hosts.^10^ Clinical analyses reveal that SARS-CoV-2 infection sustains longer with higher virus loads in severe cases compared with the mild cases, and high viral loads combined with inflammatory markers is strongly linked to the greater risk of severe outcomes or even death.^11–13^

SARS-CoV-2 infection has been associated with abnormalities in both innate and adaptive immune responses. In response to virus infection, macrophages activate inflammasomes, release cytokines, and undergo pyroptosis, contributing to the hyperinflammatory state of the lungs.^9^ The adaptive immune response plays an essential role in controlling virus infection and disease resolution, while both CD4+ and CD8 + T cells upon SARS-CoV-2 infection are characterized by dysfunctional and exhausted phenotypes and propensity for apoptosis, thereby leading to uncontrolled tissue damage and disease progression.^14–16^ Virus infection causes immune imbalance of cellular microenvironment, multi-organ damage, and cytokine storm syndrome, contributing to the development of severe COVID-19.^3,17^ It has been reported that TNF-α and IFN-γ drive inflammatory cell death, namely PANoptosis, and lethal cytokine shock by activating JAK/STAT1/IRF1 axis, which mirrors cytokine-mediated multi-organ failure.^18^ In addition, SARS-CoV-2 ORF3a facilitates HIF-1α expression by inducing mitochondrial damage and Mito-ROS production, subsequently eliciting viral infection and cytokine production.^19^ IL-2/IL-2R signal is also reported to be associated with lymphocyte decrease and critical COVID-19, which is regulated by JAK1-STAT5 pathway.^20^ The entry of SARS-CoV-2 in T helper cells leads to impaired CD4 + T cell function and may cause cell death, which is associated with viral persistence and disease severity.^21^ Our previous study demonstrates that spike protein-CD147-CyPA axis activates MAPK pathway and enhances the expression of cytokines and chemokines, which triggers the pathological changes of lung tissues in individuals with COVID-19.^22^ Hence, high virus loads and abnormal immune response are essential hallmarks for severe COVID-19 development, and investigating its underlying mechanism is of great significance for the prevention and treatment of severe COVID-19.

It is widely known that the receptors on host cells are indispensable for virus infection. High expression level of the receptors provides more channels for virus entry into host cells, which brings about extended virus infection and disease progression. As of now, angiotensin-converting enzyme-2 (ACE2), neuropilin-1, high-density lipoprotein scavenger receptor B type 1, and tyrosine-protein kinase receptor UFO are identified as important receptors or host factors for SARS-CoV-2 infection.^23–27^ Among them, ACE2, the most extensively studied receptor for SARS-CoV-2 infection, facilitates virus entry into host cells by interacting with receptor-binding domain (RBD) of the spike protein.^27,28^ However, SARS-CoV-2 infection down-regulates the membrane-bound ACE2 via ADAM17-mediated ectodomain ACE2 shedding,^29–31^ which reduces ACE2-mediated virus entry into host cells. Given the prolonged duration of virus infection and high viral loads in severe COVID-19 individuals, we speculate that some other stably expressed-receptors are responsible for continuous virus infection. In our previous studies, the cluster of differentiation 147 (CD147) is considered as a new receptor for the infection of SARS-CoV-2 and its variants by binding with RBD of the spike protein, which facilitates virus entry to host cells via Arf6-mediated endocytosis.^22,32,33^ More importantly, CD147 contributes to the tropism of SARS-CoV-2 for ACE2-deficient cell types, including T cells and astrocytes.^32,34^ It has been reported that CD147, as an inflammation-associated molecule, is markedly elevated in certain cell types of different inflammatory diseases such as atherosclerosis, rheumatoid arthritis, and psoriasis.^35–37^ CD147-mediated inflammatory reaction triggers the pathological lesions of lung tissues in COVID-19 individuals, which could be mitigated by a humanized anti-CD147 antibody, Meplazumab (MPZ).^22^ Therefore, we hypothesize that the development of severe COVID-19 is likely attributed to high CD147 expression of the host cells in the inflammatory state induced by SARS-CoV-2 infection.

In this study, we demonstrate that the CD147 receptor is inducibly expressed in host cells upon SARS-CoV-2 infection by activating the transcription factor aryl hydrocarbon receptor (AHR), which mediates the extended virus infection and immune imbalance of the hosts, leading to the pathological lesions of severe COVID-19. The administration of anti-CD147 antibody, MPZ, inhibits virus infection and cytokine level, and restores immune balance in the lung tissues of virus-infected rhesus macaque model. In addition, we resolve the structure of CD147-spike complex using cryo-EM and validate five pairs of functional residues at the interaction interface by virus infection assay and surface plasmon resonance (SPR) assay. Our findings uncover the important role of CD147 in severe COVID-19 progression from the perspective of extended virus infection and provide direct evidence for CD147-spike protein interaction, which offers new insights into the treatment of severe COVID-19.

## Results

### Blocking CD147 and ACE2 receptors inhibits virus infection and pathological lesions in a rhesus macaque model of COVID-19

**Fig. 1:**
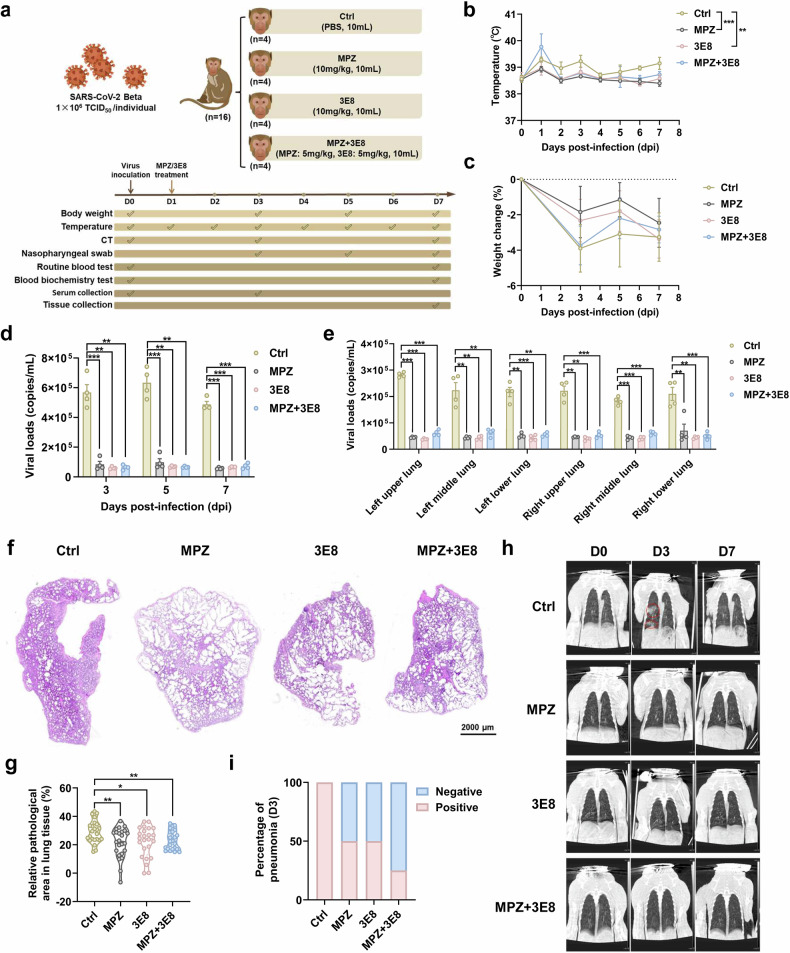
Blocking CD147 and ACE2 receptors inhibits virus infection and pathological lesions in a rhesus macaque model of COVID-19. **a** An experimental flow graph for establishing rhesus macaque model of COVID-19, *n* = 16. **b** The body temperature of rhesus macaques in four groups was detected, **p < 0.01, ***p < 0.001. **c** The body weight change of rhesus macaques in four groups were detected at 3, 5, and 7 dpi. **d** The viral loads in the nasopharyngeal swabs of rhesus macaques were analyzed at 3, 5, and 7 dpi by Taqman-based RT-PCR, **p < 0.01, ***p < 0.001. **e** The virus loads in the lung tissues of rhesus macaques were analyzed at 7 dpi by Taqman-based RT-PCR, **p < 0.01, ***p < 0.001. **f**, **g** The pathological features of lung tissues from rhesus macaques in four groups were performed by H&E staining, scale bar, 2000 μm (**f**) and the relative pathological area of lung tissues was analyzed, *n* = 24 (six masses of lung tissues from each rhesus macaque, i.e., left upper, left middle, left lower, right upper, right middle, and right lower, and four animals in each group), *p < 0.05, **p < 0.01 (**g**). **h**, **i** The chest CT tests of rhesus macaques were performed at 0, 3, 7 dpi, the abnormal inflammatory lesions in the lung tissues were indicated with red circles (**h**), the percentages of rhesus macaques with pneumonia were calculated at 3 dpi (**i**)

It has been reported that CD147 and ACE2 are two essential receptors for SARS-CoV-2 infection, and the monoclonal antibodies targeting CD147 (MPZ) and ACE2 (3E8 from Wang’s laboratory) inhibit virus entry to host cells.^27,32,38^ Primarily, the binding ability of the two monoclonal antibodies and their corresponding antigens was determined by SPR assays. The constant affinities of MPZ to human CD147 (hCD147) and 3E8 to human ACE2 (hACE2) were 0.2116 nM and 0.4585 nM; the constant affinities of MPZ to rhesus macaque CD147 (mCD147) and 3E8 to rhesus macaque ACE2 (mACE2) were 78.99 nM and 1.999 nM, indicating the two antibodies sustain a high binding ability for mCD147 and mACE2 (Supplementary Fig. 1a, b). To evaluate the efficacy of the two antibodies in COVID-19 treatment, a rhesus macaque model of COVID-19 (*n* = 16) was generated by inoculating 10^6^ TCID_50_ of the SARS-CoV-2 (Beta strain) intratracheally at 0 day post infection (dpi). Then, the randomly grouped rhesus macaques were treated with PBS (10 mL, Ctrl), MPZ (10 mg/kg in 10 mL PBS), 3E8 (10 mg/kg in 10 mL PBS), or MPZ + 3E8 (5 mg/kg MPZ and 5 mg/kg 3E8 in 10 mL PBS) at 1 dpi (Fig. 1a). Compared with the control group, the body temperature of animals decreased markedly in MPZ and 3E8 groups, and a decreasing trend was observed in MPZ + 3E8 group (Fig. 1b). Virus inoculation caused the loss of body weight, which was restored by antibody treatment in three groups (Fig. 1c). Next, we determined the viral loads in the nasopharyngeal swabs of rhesus macaques by Taqman-based RT-PCR. The animals in control group maintained a higher viral load than that in MPZ, 3E8, and MPZ + 3E8 groups at 3, 5, and 7 dpi (Fig. 1d). At 7 dpi, all rhesus macaques were euthanized to collect their lung tissues. RT-PCR results showed that the gene copies of SARS-CoV-2 decreased notably in the lung tissues of three antibody treatment groups compared with the control (Fig. 1e), which was consistent with the observation in nasopharyngeal swabs. The parameters of blood routine test and blood biochemistry test between 0 dpi and 7 dpi remained constant or fluctuated within the normal range in each group (Supplementary Fig. 2a–d). Hematoxylin & eosin (H&E) staining revealed that SARS-CoV-2 challenge caused severe inflammatory pneumonia with thickened alveolar walls, inflammatory cell infiltration, fibrinous exudate, erythrocyte diapedesis and pulmonary consolidation, which was significantly alleviated by antibody administration in three treatment groups (Fig. 1f, g and Supplementary Fig. 3a). Electron microscope further demonstrated that the pathological lesions in the lung tissues were reversed by antibody treatment (Supplementary Fig. 3b). Additionally, the apoptotic and necrotic cells and the accumulation of collagen fibrils and elastic fibers were observed in virus-infected lung tissues (Supplementary Fig. 3c). Virus-like particles were observed in AT2 cells, endothelial cells, fibroblasts, lymphocytes, macrophages, and neutrophils of virus-infected lung tissues by electron microscope (Supplementary Fig. 3d). Masson staining showed that collagen deposition relatively decreased in lung tissues of antibody treatment groups compared to the control (Supplementary Fig. 3e, f). Notably, all four rhesus macaques in the control group showed abnormal inflammatory lesions at 3 dpi using chest CT scans, whereas two in MPZ and 3E8 groups and one in MPZ + 3E8 group had pneumonia (Fig. 1h, i). Compared with 0 dpi, most cytokines, chemokines, and growth factors in serum at 3 dpi showed little change in all rhesus macaques (Supplementary Fig. 4a). Meanwhile, the level of cytokines and chemokines in lung tissues were measured and the results showed that the levels of IL-18, CCL2, CCL3, CCL8, CXCL1, CXCL9, CXCL10, and TNFα decreased markedly in MPZ group at 7 dpi compared with the control, while the levels of IL-18, CCL2, CCL3, CXCL1, and TNFα were reduced in 3E8 group, suggesting a more effective role of MPZ in inhibiting the generation of cytokines and chemokines (Supplementary Fig. 4b). These findings reveal that blocking CD147 and ACE2 receptors effectively inhibits virus infection and alleviates pathological lesions of lung tissues in COVID-19 model of rhesus macaque.

### SARS-CoV-2-induced CD147 up-regulation causes extended virus infection and pathological injury

**Fig. 2:**
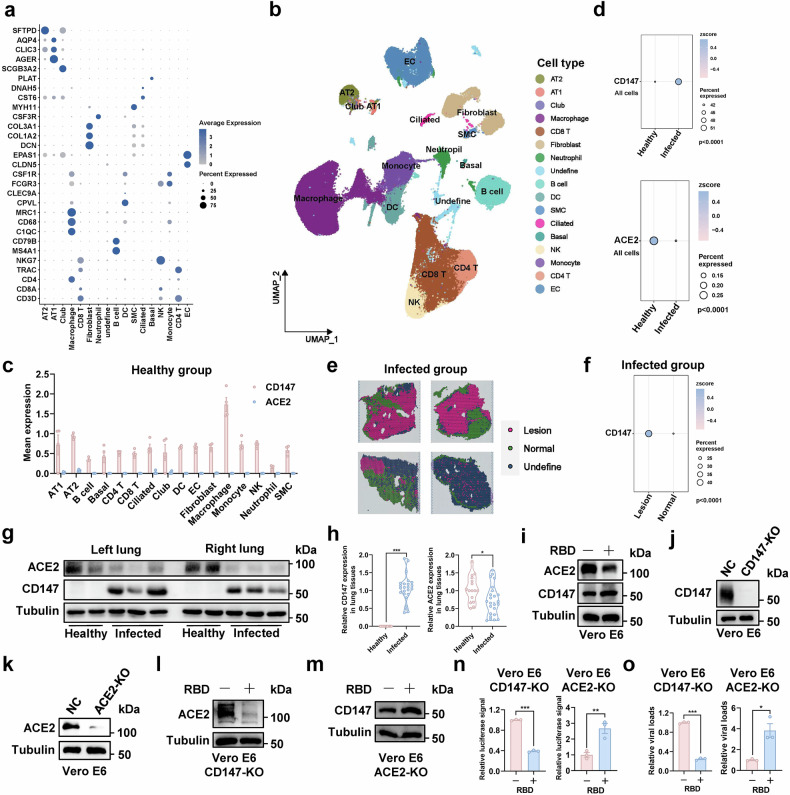
SARS-CoV-2 infection induces CD147 up-regulation coupled with down-regulated membrane-bound ACE2. **a**, **b** The clusters of scRNA-seq were identified by marker genes of corresponding cell types (**a**), and the uniform manifold approximation and projection analysis (UMAP) was presented with seventeen cell types (**b**). **c** The scRNA-seq identified the expression of CD147 and ACE2 in sixteen cell types of lung tissues from healthy rhesus macaques. **d** The expression of CD147 and ACE2 was analyzed in the lung tissues of healthy and virus-infected rhesus macaques by scRNA-seq, the cell number used for data analysis in healthy group and virus-infected group were 35,393 and 32,382, respectively. Wilcoxon test, *p* < 0.0001. **e**, **f** Normal and lesion regions in the lung tissues from virus-infected group were defined using spatial transcriptomics (**e**), and the expression of CD147 was determined in normal and lesion regions (**f**). The number of spots used for data analysis in lesion group and normal group were 3,935 and 2,468, respectively. Wilcoxon test, *p* < 0.0001. **g**, **h** The expression of CD147 and ACE2 in protein level was determined in the lung tissues from healthy and virus-infected rhesus macaques by western blot, each rhesus macaque in healthy group includes four lung samples (left, *n* = 2; right, *n* = 2), each rhesus macaque in virus-infected group includes six lung samples (left, *n* = 3; right, *n* = 3) (**g**), and the relative expression level of CD147 and ACE2 was analyzed in the lung tissues of healthy and infected groups by Image Lab software, *p < 0.05, ***p < 0.001 (**h**). **i** The expression of CD147 and ACE2 in Vero E6 cells was determined by western blot with RBD incubation. **j**, **k** The expressions of CD147 and ACE2 were detected by western blot in Vero E6 CD147-KO cells (**j**) and Vero E6 ACE2-KO cells (**k**), respectively. **l** The expression of ACE2 in Vero E6 CD147-KO cells was determined by western blot with RBD incubation. **m** The expression of CD147 in Vero E6 ACE2-KO cells was determined by western blot with RBD incubation. **n** The relative luciferase signals in SARS-CoV-2 pseudovirus-infected CD147 or ACE2 knockout Vero E6 cells were determined by dual-luciferase reporter assays with RBD incubation, **p < 0.01, ***p < 0.001. **o** The virus loads in authentic virus-infected CD147 or ACE2 knockout Vero E6 cells were determined by Taqman-based RT-PCR with RBD incubation, *p < 0.05, ***p < 0.001

Enhanced virus infection causes disease aggravation and severe COVID-19 development, which is closely associated with the receptors of host cells. To investigate the role of receptors in virus pathogenesis, scRNA-seq and spatial transcriptomics were performed on the lung tissues of virus-infected rhesus macaques (Ctrl, *n* = 4; MPZ, *n* = 4; 3E8, *n* = 4; MPZ + 3E8, *n* = 4) and healthy rhesus macaques (Healthy, *n* = 4) (Supplementary Fig. 5a). After quality control and filtering, the scRNA-seq data was clustered into seventeen cell types, and then was mapped to spatial transcriptomic data (Fig. 2a, b and Supplementary Fig. 5b, c). By analyzing the receptors for virus entry, we found that the expression of CD147 in healthy group was higher than that of ACE2 in all cell subtypes identified above (Fig. 2c), and the similar results were also obtained from spatial transcriptomics (Supplementary Fig. 5d, e). Data from scRNA-seq analysis showed that CD147 expression significantly increased in virus-infected lung tissues compared to the healthy group, while ACE2 decreased (Fig. 2d). In addition, the lung tissues in virus-infected group were defined as normal and lesion regions using spatial transcriptomics, and the analysis showed an elevation of CD147 expression in lesion region, compared to the normal region (Fig. 2e, f). The protein level of CD147 and ACE2 in lung tissues of healthy and infected groups was also determined by western blot and the results demonstrated that the expression of CD147 in protein level notably elevated in virus-infected group compared with the healthy group, while the opposite trend was observed for the expression of membrane-bound ACE2 (Fig. 2g, h). The up-regulation of CD147 and decreased membrane-bound ACE2 were also detected in Vero E6 cells incubated with spike RBD (Fig. 2i). Then, CD147 or ACE2 knockout Vero E6 cells were established by CRISPR/Cas9 system (Fig. 2j, k) and were also incubated with spike RBD protein. RBD addition induced CD147 up-regulation and enhanced the infection of authentic virus and pseudovirus in Vero E6 ACE2-KO cells, while RBD addition decreased membrane-bound ACE2 level and inhibited the infection of authentic virus and pseudovirus in Vero E6 CD147-KO cells (Fig. 2l–o). These findings demonstrate that SARS-CoV-2 infection induces CD147 up-regulation and decreases membrane-bound ACE2 level, identifying CD147 as a key contributor to extended virus infection independent of ACE2.

**Fig. 3:**
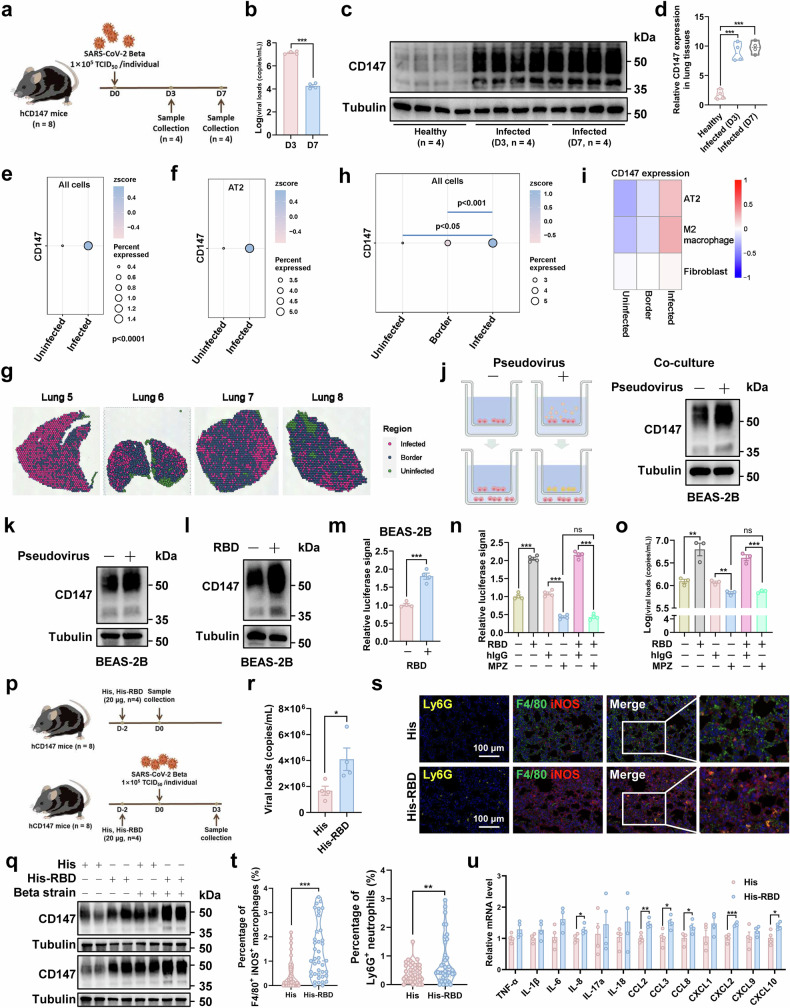
SARS-CoV-2-induced CD147 up-regulation causes extended virus infection and pathological injury. **a** A flow chart for establishing hCD147 mice model of COVID-19. **b** The virus loads in the lung tissues of hCD147 mice were detected by Taqman-based RT-PCR at 3 and 7 dpi, ***p < 0.001. **c**, **d**,The expression of CD147 was evaluated by western blot in the lung tissues of virus-infected hCD147 mice at 3 and 7 dpi, and the lung tissues of healthy hCD147 mice were used as the control (**c**). The relative expression level of CD147 was analyzed by Image Lab software, ***p < 0.001 (**d**). **e** The scRNA-seq identified the expression of CD147 in virus-infected (*n* = 4,203) and virus-uninfected (*n* = 13,354) cells of lung tissues from hCD147 mice. Wilcoxon test, *p* < 0.0001. **f** The scRNA-seq identified the expression of CD147 in virus-infected and virus-uninfected AT2 cells of lung tissues from hCD147 mice. **g–i** Infected, border, and uninfected regions in the lung tissues of virus-infected hCD147 mice were defined using spatial transcriptomics (**g**). The total expression of CD147 was quantified in three defined regions (**h**), and the expression of CD147 in AT2, M2 macrophages, and fibroblasts was determined (**i**). The number of spots used for data analysis in uninfected, border, and infected group were 312, 3,554, and 1,833, respectively. Wilcoxon test, *p* < 0.05, *p* < 0.001. **j** The BEAS-2B cells with SARS-CoV-2 pseudovirus infection in the upper chamber were co-cultured with the BEAS-2B cells in the lower chamber for 48 h, and the expression of CD147 in cells collected from the lower chamber were detected by western blot. **k**, **l** The expression of CD147 was determined by western blot in BEAS-2B cells with SARS-CoV-2 pseudovirus infection (**k**) or RBD incubation (**l**). **m** The relative luciferase signals in pseudovirus-infected BEAS-2B cells were determined by dual-luciferase reporter assays with RBD incubation, ***p < 0.001. **n** The relative luciferase signals in pseudovirus-infected BEAS-2B cells were determined by dual-luciferase reporter assays, ns, not significant, ***p < 0.001. **o** The virus loads in authentic virus-infected BEAS-2B cells were determined by Taqman-based RT-PCR, ns, not significant, **p < 0.01, ***p < 0.001. **p** A flow chart for establishing a virus-infected hCD147 mouse model by primarily inoculating with RBD protein followed by authentic virus infection. **q** The expression of CD147 was detected in the lung tissues of hCD147 mice with His-RBD (*n* = 4) or His-tag (*n* = 4) inoculation and hCD147 mice with His-RBD (*n* = 4) or His-tag (*n* = 4) inoculation followed by authentic virus infection. **r** The virus loads in the lung tissues of virus-infected hCD147 mice were determined, *p < 0.05. **s**, **t** The infiltration of F4/80+iNOS+ M1 macrophages and Ly6G+ neutrophils in lung tissues of virus-infected hCD147 mice was evaluated by multicolor immunofluorescence staining, Ly6G, yellow; F4/80, green; iNOS, red, scale bar, 100 μm (**s**), and their infiltrated percentages were quantified, **p < 0.01, ***p < 0.001 (**t**). **u** The level of cytokines and chemokines in lung tissues of virus-infected hCD147 mice was performed by RT-PCR, *p < 0.05, **p < 0.01, ***p < 0.001

Furthermore, a hCD147 mouse model of COVID-19 was established by inoculating 1 × 10^5^ TCID_50_ of SARS-CoV-2 (Beta strain) at 0 dpi. The lung tissues from 3 dpi and 7 dpi were collected to detect the viral loads and CD147 level (Fig. 3a). The results showed that the viral load was peaked at 3 dpi and decreased notably at 7 dpi (Fig. 3b). The CD147 expression in protein level markedly increased at 3 and 7 dpi (Fig. 3c, d), which was consistent with the results in rhesus macaque model. Multicolor immunofluorescence staining revealed that CD147 increased significantly in virus-infected lung tissues compared to the healthy group, the expression of CD147 in ager+ AT1 cells and sftpc+ AT2 cells also showed a marked elevation after SARS-CoV-2 infection (Supplementary Fig. 6a, b). Subsequently, AT1 and AT2 cells in lung tissues of virus-infected group were divided into virus-infected (spike + ) and virus-uninfected (spike-) types. The data demonstrated a significant elevation of CD147 expression in virus-infected AT1 and AT2 cells, compared with virus-uninfected cells (Supplementary Fig. 6c, d). ScRNA-seq and spatial transcriptomics were performed on virus-infected lung tissues of hCD147 mice at 3 dpi, the scRNA-seq data was clustered into twenty cell types, and then was mapped to spatial transcriptomic data (Supplementary Fig. 7a–d). ScRNA-seq analysis showed that the expression of CD147 was higher in virus-infected cells than that in virus-uninfected cells, including AT2 cells (Fig. 3e, f). More importantly, the virus-infected lung tissues were defined as infected, border, and uninfected regions using spatial transcriptomics, and the analysis showed that the CD147 expression increased in an order from uninfected, border, to infected regions, and this trend was also observed in AT2, M2 macrophages, and fibroblasts (Fig. 3g–i). Subsequently, the BEAS-2B cells with SARS-CoV-2 pseudovirus infection in the upper chamber were co-cultured with the BEAS-2B cells in the lower chamber for 48 h, and western blot analysis showed that the CD147 expression of BEAS-2B cells from the lower chamber was higher in SARS-CoV-2 pseudovirus group than that in the control group (Fig. 3j), suggesting an inducible elevation of CD147 in the neighboring cells of virus-infected cells. The supplement of SARS-CoV-2 pseudovirus and RBD protein enhances CD147 expression in BEAS-2B cells (Fig. 3k, l). RBD protein-induced CD147 up-regulation caused increased pseudovirus entry into BEAS-2B cells, which could be inhibited by the administration of MPZ (Fig. 3m, n). The similar results were also observed in authentic virus infection assay (Fig. 3o).

Focusing on elevated CD147, we hypothesize that SARS-CoV-2-induced CD147 up-regulation provides more channels for virus entry, contributing to severe pathological features. To test this hypothesis, we developed a mouse model of COVID-19 with RBD stimulation followed by authentic virus infection. Specifically, hCD147 mice were inoculated with RBD protein for 48 h, and His-tag was used as a control. On this basis, a hCD147 mouse model of COVID-19 (His, *n* = 4; His-RBD, *n* = 4) was generated by inoculating 1 × 10^5^ TCID_50_ of SARS-CoV-2 (Beta strain) at 0 dpi. Lung tissues were collected to detect the expression of CD147 at different time points (Fig. 3p). Compared to the His group, the level of CD147 increased notably in RBD group, which was further intensified with authentic virus infection (Fig. 3q). The virus loads increased significantly in the lung tissues of RBD group compared to the control group (Fig. 3r). The infiltration of main immune cells in lung tissues increased significantly, including F4/80+iNOS+ M1 macrophages and Ly6G+ neutrophils (Fig. 3s,t). Compared with the control, the level of cytokines and chemokines in lung tissues elevated in RBD group, including IL-8, CCL2, CCL3, CCL8, CXCL2, and CXCL10 (Fig. 3u). Taken together, these findings indicate that SARS-CoV-2 infection facilitates the inducible up-regulation of CD147, which enhances the susceptibility of host cells to virus and enables extended virus infection, contributing to severe pathological lesions in lung tissues.

### SARS-CoV-2-induced CD147 expression is regulated by transcription factor AHR

**Fig. 4:**
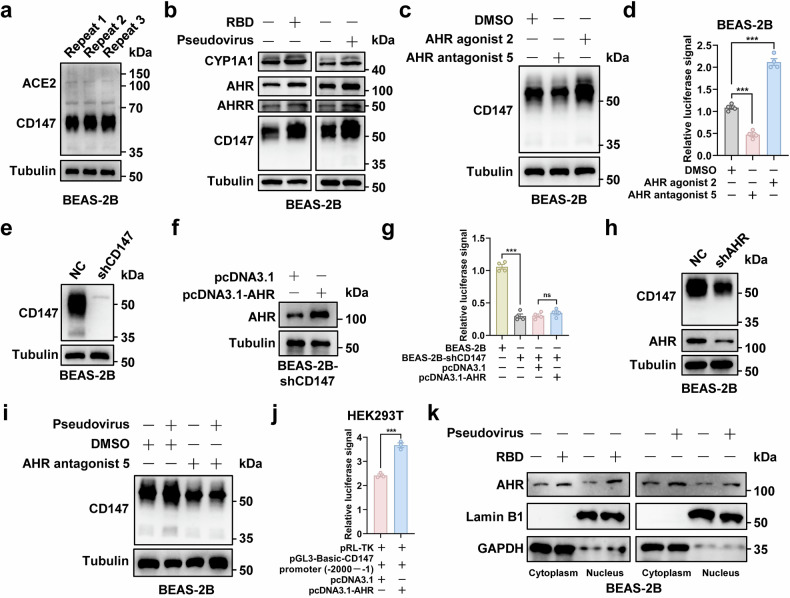
SARS-CoV-2-induced CD147 expression is regulated by transcription factor AHR. **a** The expressions of CD147 and ACE2 in BEAS-2B cells were determined by western blot. **b** The expressions of CD147, AHR, AHRR, and CYP1A1 in BEAS-2B cells were determined by western blot with SARS-CoV-2 pseudovirus infection or RBD incubation. **c** The expression of CD147 was determined in BEAS-2B cells by western blot with the addition of AHR agonist 2 or AHR antagonist 5. **d** The relative luciferase signals in pseudovirus-infected BEAS-2B cells were determined by dual-luciferase reporter assays with the addition of AHR agonist 2 or AHR antagonist 5, ***p < 0.001. **e** The expression of CD147 in BEAS-2B-shCD147 cells and its control was determined by western blot. **f** The transfection efficiency of plasmid AHR was evaluated by western blot in BEAS-2B-shCD147 cells. **g** The relative luciferase signals were determined by dual-luciferase reporter assays in pseudovirus-infected BEAS-2B cells and BEAS-2B-shCD147 cells with or without the transfection of plasmid AHR, ns, not significant, ***p < 0.001. **h** The expression of CD147 was determined in AHR knockdown BEAS-2B cells by western blot. **i** The expression of CD147 was determined in pseudovirus-infected BEAS-2B cells by western blot with the addition of AHR antagonist 5. **j** Dual-luciferase reporter assays were performed after co-transfecting of the pGL3-Basic-CD147 promoter (−2000– − 1), pRL-TK, and plasmid AHR into HEK293T cells. The relative luciferase signal was detected using a Dual-Luciferase® Reporter Assay System, ***p < 0.001. **k** Nuclear and cytoplasmic protein extraction assays were performed to determine the nuclear expression and cytoplasmic expression of AHR in BEAS-2B cells with pseudovirus infection or RBD incubation. Lamin B1 and GAPDH were selected as controls for nuclear protein and cytoplasmic protein, respectively

The above results show that SARS-CoV-2 infection elicits CD147 up-regulation and causes extended virus infection and pathological injury. However, the mechanism for virus-induced CD147 up-regulation remains unclear. Previous studies show that AHR, a transcription factor, is expressed widely in pulmonary epithelial cells and regulates the inflammatory response to microorganisms, including viruses, bacteria, parasites, and fungi.^39^ For SARS-CoV-2, the activation of AHR signaling facilitates virus replication and exacerbates lung pathology.^40,41^ Hence, we investigated whether virus-induced CD147 up-regulation is associated with AHR activation. BEAS-2B cells that express CD147 but do not express ACE2 were used to test the hypothesis (Fig. 4a). Firstly, the expressions of AHR, CD147, and the target genes regulated by AHR, including AHRR and CYP1A1,^42,43^ were determined by western blot in BEAS-2B cells after the addition of SARS-CoV-2 pseudovirus and RBD protein. The results showed that pseudovirus and RBD protein enhanced CD147 expression in BEAS-2B cells, as well as AHR, AHRR, and CYP1A1 (Fig. 4b). The addition of AHR antagonist 5 inhibited the expression of CD147 in BEAS-2B cells, and the opposite results were obtained after adding AHR agonist 2 (Fig. 4c). Meanwhile, SARS-CoV-2 pseudovirus infection assay showed that the addition of AHR antagonist 5 weakened pseudovirus entry into BEAS-2B cells owing to the decreased expression of CD147, while the enhanced pseudovirus infection was observed in the group with the administration of AHR agonist 2 (Fig. 4d). Then, we established a CD147-knowndown cell line, BEAS-2B-shCD147, by lentivirus infection (Fig. 4e). After being transfected with plasmid AHR, BEAS-2B-shCD147 cells were infected with SARS-CoV-2 pseudovirus. Compared to the control, the luciferase signal decreased significantly in CD147 knockdown group, which could not be reversed by AHR overexpression (Fig. 4f, g). Subsequently, AHR knockdown cell line was generated using lentivirus infection, and the results showed that the silencing of AHR reduced CD147 expression in BEAS-2B cells (Fig. 4h). Pseudovirus infection-induced CD147 up-regulation was suppressed by the addition of AHR antagonist 5 (Fig. 4i). In addition, the dual-luciferase reporter assay showed that the CD147 gene promoter exhibited a greater luciferase signal in AHR group than in the control group (Fig. 4j). Pseudovirus infection and RBD protein stimulation enhanced AHR expression and facilitated its translocation to the nucleus in BEAS-2B cells (Fig. 4k). These findings indicate SARS-CoV-2-induced CD147 expression is regulated by transcription factor AHR, which contributes to extended virus infection.

### Immune imbalance caused by SARS-CoV-2 infection is restored by blocking CD147 receptor

**Fig. 5:**
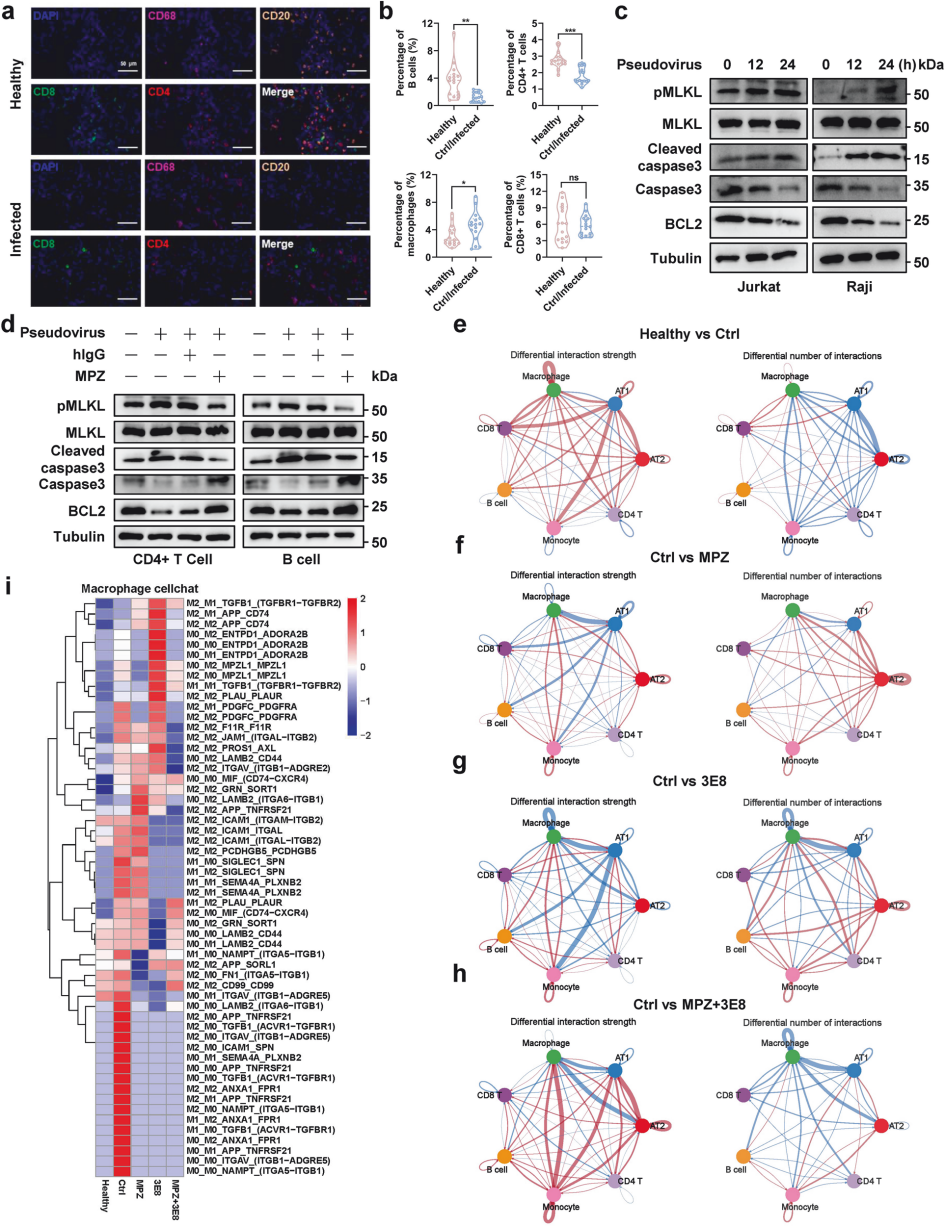
Immune imbalance caused by SARS-CoV-2 infection is restored by blocking CD147 receptor. **a**, **b** Multicolor immunofluorescence staining was performed to detect the infiltration of CD68+ macrophages, CD20 + B cells, CD8 + T cells, and CD4 + T cells in the lung tissues of healthy and virus-infected rhesus macaques, scale bar, 50 μm (**a**), and the cell percentages were analyzed in the two groups, ns not significant, *p < 0.05, **p < 0.01, ***p < 0.001 (**b**). **c** The expressions of BCL2, caspase3, cleaved caspase3, MLKL, and p-MLKL were detected by western blot in Jurkat and Raji cells with pseudovirus infection for 0, 12, and 24 h. **d** The expressions of BCL2, caspase3, cleaved caspase3, MLKL, and p-MLKL were detected by western blot in CD4 + T and B cells treated with MPZ followed by pseudovirus infection. **e–h** The cell-cell communications were performed by CellChat using data from scRNA-seq of rhesus macaques, including Healthy vs Ctrl (**e**), Ctrl vs MPZ (**f**), Ctrl vs 3E8 (**g**), and Ctrl vs MPZ + 3E8 (**h**). **i** The cell-cell communications were performed by CellChat among M0, M1, and M2 macrophages, and the receptor-ligand interactions were presented among the five groups of rhesus macaques

Immune damage caused by SARS-CoV-2 is an important contributor to severe COVID-19 progression.^44^ Hence, we analyzed the abnormal changes of cell composition in the lung tissues of rhesus macaques. ScRNA-seq showed that the percentages of main immune cells, including B cells, CD4 + T cells, and CD8 + T cells, displayed a decreasing trend in the virus-infected group, compared to the healthy group, while the percentages of macrophages increased (Supplementary Fig. 8a). Then, the above-mentioned four types of immune cells in lung tissues were determined by multicolor immunofluorescence staining (Fig. 5a), and the results demonstrated a decreasing trend in the percentages of CD4 + T cells and B cells of the virus-infected group compared to the healthy group, the percentage of macrophages increased, and the percentage of CD8 + T cells remained unchanged in the lung tissues after virus infection (Fig. 5b). To explore the rationale for the decreased CD4 + T cells and B cells in virus-infected tissues, the scRNA-seq data from the lung tissues of rhesus macaques was used to analyze the expression levels of cell death markers in CD4 + T cells and B cells. The results showed that the gene expressions of BAX, BAX/BCL2, BAK1, TNFSF10, FADD, and MLKL exhibited an increased trend in both CD4 + T cells and B cells of virus-infected group compared to the healthy group (Supplementary Fig. 8b, c), which indicates that virus infection may promote the apoptosis and necroptosis of CD4 + T cells and B cells. Next, the signature signals of apoptosis and necroptosis were determined using Jurkat and Raji cells upon pseudovirus infection. Western blot showed that the expression of anti-apoptotic protein BCL2 decreased in a time-dependent manner after pseudovirus infection, while the level of cleaved caspase3 and pMLKL increased in Jurkat and Raji cells (Fig. 5c). The similar results were also observed in pseudovirus-infected CD4 + T and B cells isolated from human peripheral blood mononuclear cells, which could be reversed by the administration of MPZ (Fig. 5d and Supplementary Fig. 8d, e). These findings reveal that CD147 receptor-mediated SARS-CoV-2 infection induces the apoptosis and necroptosis of CD4 + T and B cells, leading to the destruction of immune homeostasis.

Cell-cell communication is of great importance for normal physiological processes, including body growth and development, and its imbalance is likely to cause the abnormalities in biological functions and disease progression.^45^ The cell communication among the major cell types in lung tissues, including AT1, AT2, B cells, CD4 + T cells, CD8 + T cells, monocytes, and macrophages, was analyzed by CellChat. The results showed that the interaction strength and interaction numbers of macrophage-macrophage communication was significantly enhanced in SARS-CoV-2-infected group compared to that in the healthy group (Fig. 5e), which could be restored by MPZ and 3E8 treatment (Fig. 5f–h). Then, we re-clustered the macrophages into three subtypes, namely M0, M1, and M2 macrophages (Supplementary Fig. 8f, g). Although the percentages of three subtypes remained relatively consistent across five groups (Supplementary Fig. 8h), the cell-cell communications exhibited abnormal changes, especially for M2 macrophage communications, which were closely associated with immunosuppression. Blocking CD147 and ACE2 receptors by MPZ and 3E8 restored the dysregulated macrophage communications, suggesting an enhanced antiviral capacity in macrophages (Fig. 5i). In addition, scRNA seq analysis of lung tissues from hCD147 mice also showed a stronger cell-cell communication among M2 macrophages in virus-infected group, compared to the virus-uninfected group (Supplementary Fig. 9a, b). We also analyzed the cell-cell communication between M2 macrophages and other cell types in the lung tissues of virus-infected hCD147 mice. The results showed that M2 macrophages had stronger capacity to communicate with virus-infected AT2 cells and CD8 + T cells, compared to the virus-uninfected cells (Supplementary Fig. 9c, d). These findings reveal that SARS-CoV-2 infection contributes to abnormal cell-cell communications among different cell types, especially for M2 macrophages, and inhibiting virus infection by blocking CD147 and ACE2 receptors plays an important role in maintaining cell communication homeostasis, which provides a promising intervention strategy for severe COVID-19.

### Cryo-EM structure demonstrates CD147-spike interaction

**Fig. 6:**
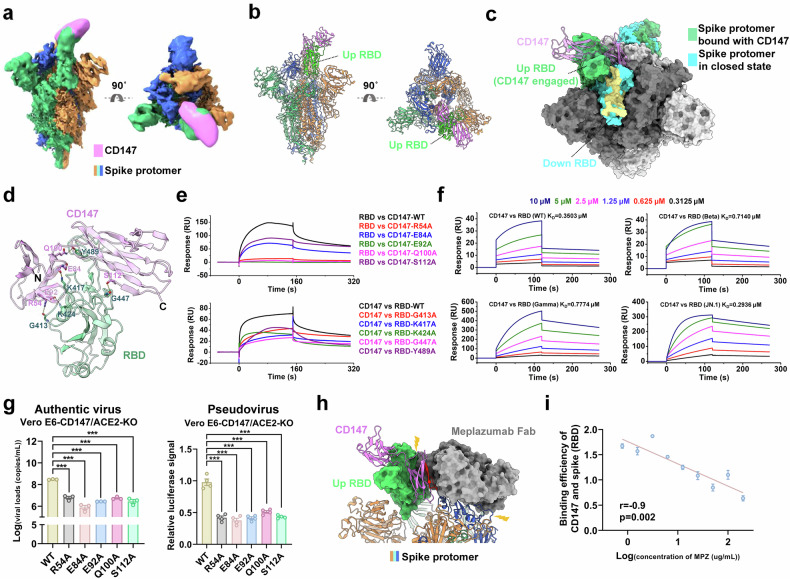
Cryo-EM structure demonstrates CD147-spike interaction. **a** Cryo-EM map of the CD147-spike complex. The RBD up protomer, green; the two RBD down protomers, sandy brown and blue; CD147, magenta. **b** The overall structure of the CD147-spike complex shown as cartoons in side (left) and top (right) views. **c** The overlaid structures of CD147-spike and spike-closed are shown in surface representation. CD147 is depicted in magenta, with the spike protomer in the up and down conformations shown in green and cyan, respectively. The contact patch recognized by CD147 is highlighted in yellow on the down RBD. **d** The structure and key residues within the binding interface of the CD147-spike complex. The key residues forming hydrogen bonds are presented by stick models, with the hydrogen-bonding interactions labeled in yellow lines. **e** The binding ability of spike RBD with wildtype CD147 or its mutants (CD147-R54A, E84A, E92A, Q100A, and S112A) and CD147 with wildtype spike RBD or its mutants (G413A, K417A, K424A, G447A, and Y489A) was determined by SPR assay. **f** The binding ability of CD147 with wildtype spike RBD or its mutants (spike RBD-Beta, Gamma, and JN.1) was determined by SPR assay. **g** The virus loads and relative luciferase signals in CD147/ACE2 double knockout Vero E6 cells transfected with either wild type CD147 or mutated CD147 were determined by Taqman-based RT-PCR and dual-luciferase reporter assays, respectively, ***p < 0.001. **h** The overlaid structures of the CD147-spike complex, colored as in (a), and the CD147-Meplazumab Fab complex (PDB ID: 5X0T), colored in gray. The up RBD and Meplazumab Fab are depicted as surfaces, with the binding epitopes on CD147 (residues 61-75, colored in red) recognized by Meplazumab. The steric hindrance between Meplazumab and the spike bound to CD147 is indicated with orange arrows. **i** The competitive inhibitory role of MPZ for the binding of CD147 and SARS-CoV-2 spike (RBD) protein was determined by ELISA assay

Previous study views CD147 as a receptor for SARS-CoV-2 infection, which facilitates virus entry into host cells by binding with spike protein.^32^ However, the intricate process of virus recognition and infection remain unclear. In our study, we conducted imaging of individual particles of the spike protein incubated with an excess of the CD147 extracellular cellular domain (CD147-ECD) using cryo-EM (Supplementary Fig. 10a). The 2D and focused 3D classification revealed the coexistence of the CD147-spike complex and unbound spike trimers in three distinct states: spike closed, spike with one RBD up, and spike with two RBD up (Supplementary Fig. 10b–d). A well-defined 3D class comprising 62,906 particles enabled the reconstruction of the complex of CD147-bound RBD in the up conformation of spike trimer at a resolution of 3.75 Å (Fig. 6a and Supplementary Fig. 10d, e). The atomistic model of the CD147-spike complex, refined by molecular dynamics (MD) simulations to characterize local conformational flexibility,^46^ exhibited strong agreement with the cryo-EM density map (Fig. 6b and Supplementary Fig. 10f, g).

By superimposing the structure of the CD147-spike complex with the closed state of the spike trimer, we found that CD147-recognition motif within the RBD was buried at the interface between protomers in the closed conformation of the spike trimer (Fig. 6c). The upward RBD undergoes a 32.3° rotation, both upward and outward, exposing the receptor-binding motif (RBM) and making it accessible for CD147 binding (Fig. 6c and Supplementary Fig. 10 h). Furthermore, we observed significant clockwise rotations in the N-terminal domains (NTDs) of the three protomers upon binding with CD147 (Supplementary Fig. 10i). The CD147-spike structure reveals that CD147 binding to SARS-CoV-2 necessitates the up conformation of the RBD and may induce conformational dynamics within the spike.

Cryo-EM reconstruction demonstrated that the loop connecting two IgG domains of CD147 straddles the β6 strand (residues 490-493) of the RBD, creating a concave binding surface that comfortably accommodates CD147. Analysis of the interface between the spike and CD147 showed that residues R54, E84, E92, Q100, and S112 of CD147 form hydrogen bonds with residues G413, K417, K424, Y489, and G447 of the spike, respectively (Fig. 6d). To further validate the role of these residues, we mutated each residue to alanine (A) and conducted SPR assays, which demonstrated weaker affinity between the RBD and the mutated CD147 proteins compared to the wild type, and similarly, between CD147 and the mutated RBD proteins (Fig. 6e). Among them, K417 of the spike is a hot-spot mutant in SARS-CoV-2 variants, such as K417N in SARS-CoV-2 Beta and Omicron strains and K417T in Gamma Strain. However, the SPR assays demonstrated that the mutation K417N/T had little or slight impact on the interaction between the CD147 and RBD of Beta, Gamma, and JN.1 strains (Fig. 6f), which was also identified by the calculation of the binding free energies of CD147 with spike RBD and its variants (Supplementary Table 1). Virus infection assays using CD147/ACE2 double knockout Vero E6 cells showed that the mutated CD147 significantly inhibited authentic virus entry into host cells compared to the wild type CD147, which was also verified in SARS-CoV-2 pseudovirus infection tests (Fig. 6g). In addition, we performed structural alignment of the CD147-spike complex with the previously reported CD147-MPZ Fab complex (PDB ID: 5X0T),^47^ and the results indicated extensive clashes between the binding interfaces of MPZ and the spike RBD with CD147 (Fig. 6h). Competitive inhibition assay demonstrated that MPZ directly blocked the binding of spike RBD to CD147 in a dose-dependent manner (Fig. 6i). Collectively, these results provide structural insights into how MPZ inhibits virus infection by disrupting the CD147-spike interaction. In summary, our findings present cryo-EM structures of CD147-spike complex and identify the key amino acid residues at the interaction interface between the two proteins, providing direct evidence for SARS-CoV-2 infection via the CD147 receptor.

## Discussion

Up to now, severe COVID-19 remains a major cause of high mortality among hospitalized individuals and pose significant threat to the public health and global economy. Due to the limited strategies in clinical management, blocking the receptors for virus infection has aroused wide attention in severe COVID-19 treatment. Our previous clinical trials demonstrate that Meplazumab targeting CD147 receptor exhibits favorable safety and efficacy in treating severe COVID-19, which significantly reduces the mortality, the viral loads, and the cytokine levels.^48,49^ Here, we discovered that CD147 was inducibly up-regulated upon virus infection via AHR activation, which boosted extended virus infection and immune imbalance, resulting in serious pathological lesion in lung tissues. These findings unveil the pathogenesis of severe COVID-19 progression. Meanwhile, the determination of the CD147-spike complex structure provides solid evidence that CD147 is a receptor engaging ACE2-independent SARS-CoV-2 cell entry. Additionally, humanized antibody targeting CD147, Meplazumab, directly disrupts the interaction between CD147 and spike protein, which renders a firm support for its clinical practice in severe COVID-19 treatment.

It has been reported that AHR is a cytosolic transcription factor activated by corresponding ligands such as kynurenine. Once the AHR is activated, it trans-locates to the nucleus and then dimerizes with the AHR nuclear translator to promote gene transcription.^50,51^ In SARS-CoV-2 infection, AHR activation exerts an important influence on the innate immune response regulated by neutrophils, macrophages, and mast cells, and adaptive immune response regulated by CD8 + T cells, which causes imbalanced immune response and severe COVID-19.^52^ In our study, we identified CD147 gene as a new target of AHR that regulated inducible CD147 expression in virus-infected cells, and further amplified CD147 expression in neighboring virus-free cells, providing more “doors” for virus entry.

Compared to CD147, the membrane-bound ACE2 decreases in SARS-CoV-2 infection due to ADAM17-mediated ectodomain ACE2 shedding,^29–31^ which causes gut dysbiosis, inflammation, coagulopathy, and damage to cardiovascular system and kidneys owing to the loss of its protective functions in various organs.^31,53,54^ These findings demonstrate that the changes of CD147 and ACE2 expression caused by SARS-CoV-2 infection contribute to severe COVID-19 progression from two different perspectives, and the inducible expression of CD147 is an important driver for severe COVID-19, which provides a reasonable explanation for the high viral load in severe COVID-19.

Dysregulated immune response is a key hallmark in SARS-CoV-2 infection, which manipulates the progression of severe COVID-19. SARS-CoV-2 virions evade the innate immune system through multiple strategies, which are closely related to the activation of TLR, PRR, and IFN signalings.^17,55,56^ The destruction of adaptive immunity suppresses the function of B cells and T cells, including the production of neutralizing antibodies and the persistence of immune memory.^57^ In our study, SARS-CoV-2-infected rhesus macaque model demonstrated the immune imbalance characterized by decreased CD4 + T cells and B cells in the lung tissues, which was closely associated with cell apoptosis and necroptosis. Importantly, the administration of MPZ reversed the cell death signals of CD4 + T cells and B cells. In contrast to ACE2, which is not expressed by CD4 + T cells and B cells, CD147 is widely expressed in the two types of cells and responsible for direct SARS-CoV-2 infection, which is prone to promote immune imbalance and consequent severe pathological injury in the lung tissues of COVID-19 individuals. Our findings provide a unique insight into the role of MPZ in treating severe COVID-19. Apart from T and B cells, macrophages, a highly infiltrating cell type in lung tissues, also play an important role in regulating abnormal immune response. The activated interstitial macrophages are identified as key contributors to inflammation and fibrosis in early COVID-19,^58^ the interplay between macrophages and NK cells regulate SARS-CoV-2 persistence in macrophages.^59^ In our study, we found that SARS-CoV-2 infection elicited abnormal M2 macrophage communications, contributing to immunosuppression in virus-infected lung tissues. The administration of MPZ and 3E8 restored the immunosuppressive microenvironment caused by virus infection. These findings indicate that the intervention by receptor blockades activates macrophage-mediated innate immunity and improves virus-mediated immune imbalance. Data from hCD147 mice model demonstrated the enhanced communications between M2 macrophages with virus-infected AT2 cells and CD8 + T cells, compared to the virus-uninfected cells. These findings indicate M2 macrophages play a dominant role in causing immune imbalance by affecting cell communications upon SARS-CoV-2 infection. This highlights the critical importance of restraining macrophage-mediated pathological immune responses in severe COVID-19.

Our study had resolved the cryo-EM structure of the CD147-spike complex. Similar to ACE2, CD147 exclusively binds to the erect RBD of the spike protein in its open conformation, inducing significant clockwise rotations in the NTDs of the three protomers. Therefore, CD147 binding to spike may shift the conformational landscape of the spike trimer towards more open states. The transition of the spike protein from the RBD-closed to the RBD-up conformation might trigger considerable conformational dynamics in the S1 subunits and mobilize the fusion peptide, thereby priming the spike protein for membrane fusion.

Cryo-EM analyses and MD simulations identified the receptor-binding motif of the RBD as the CD147-binding platform. The significant overlap in the binding footprints of CD147 and ACE2^60^ on the RBD suggests that these receptors cannot bind to the spike protein simultaneously (Supplementary Fig. 10j), indicating that CD147 and ACE2 represent two independent routes for SARS-CoV-2 invasion. The binding interface shared by CD147 and ACE2 is partially buried between adjacent protomers in the closed conformation of the spike trimer. This structural feature may reveal a sophisticated mechanism by which SARS-CoV-2 evades humoral immunity prior to cell entry.

Our previous study identifies CD147 as a universal receptor for the infection of SARS-CoV-2 and its variants.^22^ The findings from SPR and binding free energy calculations showed CD147 could bind with the spike RBD of Beta, Gamma, and Omicron (JN.1), which reinforces our conclusions. Based on the structure of CD147-spike complex, we identified five pairs of key residues mediating the CD147-spike interaction. Notably, the Gamma variant carrying the K417T mutation exhibited a slightly decreased affinity for CD147, consistent with binding free energy calculations based on the CD147-spike complex structure. This mutation is also reported to decrease the affinity of the spike protein for ACE2.^61^ The persistence of the K417T mutation in the spike protein may be attributed to its ability to mediate immune escape while maintaining relatively high affinity for CD147 and ACE2.^62^

In summary, this study reveals that inducible CD147 up-regulation boosts extended SARS-CoV-2 infection, emphasizing the crucial role of CD147 in facilitating severe COVID-19.

## Materials and methods

### Ethics statement

All the authentic virus studies were performed in biosafety level 3 facility. The procedures and methods in in-vivo test involving rhesus macaque and mice were approved by the Institutional Animal Care and Use Committee of the National Translational Science Center for Molecular Medicine, Fourth Military Medical University (2023-NTSCMM-ID010), Guangxi Medical University (202303038), and the Institute of Laboratory Animal Science, Peking Union Medical College (BLL23002). The human sample (peripheral blood mononuclear cells) collection was approved by the ethics committee of Xijing Hospital, Fourth Military Medical University (KY20244099-1).

### Expression and purification of SARS-CoV-2 spike trimer and CD147

A mammalian codon-optimized DNA fragment encoding the ectodomain region (residues 1 − 1208) of the stabilized trimeric SARS-CoV-2 spike protein (GenBank: MN908947), with two proline substitutions and a modified furin cleavage site, was cloned into the pcDNA3.4(+) vector. This construct included a C-terminal T4 fibritin trimerization motif and a Flag tag. The vector was transiently transfected into FreeStyle 293 F cells (ThermoFisher Scientific) using polyethylenimine. The spike protein was purified from filtered cell supernatants using anti-Flag affinity resin (GenScript), followed by size-exclusion chromatography on a Superdex 200 10/300 Increase column (GE Healthcare) in PBS at pH 7.4.

For the expression of CD147 and its mutants, the DNA fragment encoding CD147-ECD (residues 22 − 205) with a C-terminal His-tag was codon-optimized and inserted into the pET21a(+) vector. This construct was expressed in E. coli Origami B (DE3) cells. CD147 was purified using Ni2+ affinity chromatography, followed by anion-exchange chromatography and gel filtration chromatography, as previously described.^47^ Both purified proteins were concentrated, flash-frozen in liquid nitrogen, and stored at −80 °C for subsequent cryo-EM and SPR experiments.

### Cryo-EM sample preparation, data collection and processing

To obtain the CD147-spike complex, 3.5 μL aliquots of the CD147-spike mixture was loaded onto a freshly glow discharged lacey carbon grid (Quantifoil Au 300 mesh, R1.2/1.3) with a thin layer of evaporated continuous carbon prior to plunge freezing using a vitrobot MarkIV (ThermoFisher Scientific) at 4 °C and with 100% humidity. Samples were then transferred to an FEI Titan Krios transmission electron microscope operated at 300 kV and equipped with a Falcon III Summit direct detector and Gatan Quantum GIF energy filter, operated in zero-loss mode with a slit width of 20 eV. Automated data collection was carried out using EPU at a nominal magnification of 96,000× with a pixel size of 0.846 Å. Each movie was collected in super-resolution mode fractionated for 40 sec with a total of 35 frames with the dose rate of 1.53 e^−^/Å^2^ per frame. 2,402 micrographs were collected in a single session with a defocus range comprised between –1.2 and –2.5 μm.

All image processing was executed using RELION-3.1.2.^63^ All images were summed and motion-corrected using MotionCorr2.^64^ Undesirable pictures contaminated by crystalline ice or other visible forms of contamination were removed by manual inspection and micrographs with maximum estimated resolution beyond 4.0 Å were discarded after the contrast transfer function (CTF) parameters were estimated by GCTF.^65^ A total of 641,033 particles were auto-picked by Gautomatch with reference to the 2D classification of the manually picked particles. Particles extracted with a box size of 330 pixels from the dataset were subjected to multiple iterations rounds of reference-free 2D classification to remove junk classes leaving approximately 505,182 good particles. Wherein 189,656 particles selected for the CD147-spike complex were used to determine the initial models and were 3D classified with 3 classes. One good class was selected for further 2D classification and 3D classification. A dataset of 62,906 were subjected to 3D auto-refinement, resulting in an initial 4.65 Å density map. Further Bayesian polishing and auto-refinement of these particle projections was performed to generate a final 3.75 Å map determined by gold standard Fourier shell correlation (FSC) using the 0.143. Local resolution estimation was performed in RELION-3.1.2 using the unfiltered half map. For the unbound spike trimer structure in different states, 315,526 particles were extracted from the 505,182 good particles obtained in the 2D classification for further 3D classification and we obtained 3 different conformational subclasses with two RBDs open state, one RBD open state and closed state. 56,015 particles in two RBDs open state were further applied for final homogeneous refinement in RELION-3.1.2 and obtained the best density map with global resolution of 4.07 Å. 122,316 particles in one RBD open state were further applied for final homogeneous refinement in RELION-3.1.2 and obtained the best density map with global resolution of 3.36 Å. 46,532 particles in RBD closed state were further applied for final homogeneous refinement in RELION-3.1.2 and obtained the best density map with global resolution of 3.25 Å.

### Protein model construction and molecular dynamics (MD) simulation

The initial coordinates of CD147-SARS-CoV-2 spike were conducted with the structures spike (7DDN) and CD147 (3B5H), and were fitted into the 3.75 Å post-processing map by using UCSF Chimera^66^ and molecular dynamics flexible fitting (MDFF) protocol,^67^ in order to maximize the correlation coefficient between atomic coordinates and the density map (9UG3, Supplementary Table 2).^68^ The adjustment structure was subsequently refined by the 100.0 ns MD simulations with the position constraint by the density map.^69^ System setup of each MD simulation is agreed with previous studies.^70,71^ Coordinate values were collected every 10 ps for density cross-correlation analysis to avoid any potential model overfitting during refinement. The binding free energies (*ΔG*_*bind*_) of CD147 with SARS-CoV-2 spike RBD and its variants were calculated by the molecular mechanics generalized Born surface area (MM/GBSA) approach in AMBER Tools 19,^72^ with 100 snapshots evenly extracted from the MD trajectories. All figures were presented using Chimera,^66^ and the analysis of binding interface between CD147 and SARS-CoV-2 spike was conducted based on the MD refined structures.

### SARS-CoV-2 virus

Authentic SARS-CoV-2 viruses for cell assays were supplied by Chinese Center for Disease Control and Prevention, including original and Beta strains. The authentic SARS-CoV-2 virus (Beta variant) for in-vivo assays was supplied by Institute of Laboratory Animal Science, Chinese Academy of Medical Sciences and Comparative Medicine Center, Peking Union Medical College. All the authentic viruses were determined using a standard TCID_50_ assay. SARS-CoV-2 pseudoviruses, including original and Beta strains, were kindly provided by Institute of Medical Biology, Chinese Academy of Medical Science and Peking Union Medical College.

### Cell lines

BEAS-2B, Vero E6, HEK293T, Jurkat, and Raji cells were obtained from the Cell Bank of the Chinese Academy of Sciences, which were authenticated using Short Tandem Repeat DNA profiling (Beijing Microread Genetics). Generally, all the cells were cultured at 37 °C under 5% CO_2_ in DMEM or RPMI 1640 medium supplemented with 10% fetal bovine serum (FBS) and 2% L-glutamine. The gene knockout cells (Vero E6 CD147-KO, Vero E6 ACE2-KO, Vero E6 CD147/ACE2-KO) were generated using CRISPR/Cas9 system (GeneChem). BEAS-2B cell was used to generate AHR knockdown cell line by transfecting the lentivirus encoding the shAHR constructs (L00117, Beyotime).

### SARS-CoV-2 infection assays

The cells (Vero E6-CD147-KO and Vero E6-ACE2-KO) were cultured in 24-well plates at 37 °C overnight. His-RBD protein (500 ng/mL, 40592-V08H, Sino Biological) and 6×His-tag (500 ng/mL, QYAOBIO) were added in the medium for 24 h. Then, the cell medium was discarded and the authentic SARS-CoV-2 virus (Beta, 100 TCID_50_) was added into each well for 2 h. Subsequently, the cell supernatant was replaced with 2% FBS medium and the cells were cultured at 37 °C for 48 h. Finally, the samples were collected and the virus loads were detected by Taqman-based real-time PCR. For the key residue identification between CD147 and spike RBD, the plasmids encoding wild type CD147 and its mutants, including CD147-R54A, CD147-E84A, CD147-E92A, CD147-Q100A, and CD147-S112A, were transfected into Vero E6 CD147/ACE2-KO cells, using a versatile DNA/siRNA transfection reagent (PT-114-15, Polyplus), respectively. Then, the cells in different groups were infected with authentic SARS-CoV-2 virus (original strain) and the virus loads were detected following the above procedures. For BEAS-2B cells, the cells were incubated with His-RBD protein (500 ng/mL, 40592-V08H, Sino Biological) and 6×His-tag (500 ng/mL, QYAOBIO) for 24 h. Then, the cell medium was replaced with 2% FBS medium containing hIgG (30 μg/mL, I4506, Sigma-Aldrich) and MPZ (30 μg/mL, Jiangsu Pacific Meinuoke Biopharmaceutical Co., Ltd.). After 1 h incubation, the cells were infected with the authentic SARS-CoV-2 virus (Beta, 100 TCID_50_) for 2 h. Subsequently, the supernatant was discarded and the medium containing hIgG (30 μg/mL, I4506, Sigma-Aldrich) and MPZ (30 μg/mL) was added into the corresponding wells for 48 h. The samples were harvested to detect the virus loads. The same procedures were applied in pseudovirus infection assay, with the infection efficiency evaluated by detecting firefly fluorescence signal using Dual-Luciferase Reporter Assay System (E1980, Promega) according to the manufacturer’s protocols. AHR agonist 2 (HY-144339, MCE) and AHR antagonist 5 (HY-141609, MCE) are potent agonist and inhibitor of AHR, respectively, which can regulate the transcription of AHR’s downstream genes by affecting its nuclear localization. The concentration of AHR agonist 2 and AHR antagonist 5 used in cell tests was 100 nM.

### ELISA

ELISA assay was performed to detect the competitive inhibitory effect of MPZ for the binding of CD147 and SARS-CoV-2 spike RBD protein. The His-CD147 protein (5 μg/mL, our laboratory) was coated on microplate at 4 °C overnight. After blocked with 1% BSA for 1 h, the wells were incubated with spike RBD (25 μg/mL, GenScript) and MPZ (two-fold dilution, 200–0.781 μg/mL) at 37 °C for 1 h. After washed with PBST, the samples were incubated with anti-spike RBD antibody (2 μg/mL, 40591-MM43, Sino Biological) and goat anti-mouse IgG (H + L) secondary antibody (31430, ThermoFisher Scientific) in sequence. After coloration, the OD value at 450 nm was measured with microplate reader (BioTek). The cytokines, chemokines, and growth factors in serum of rhesus macaques were also detected using ProcartaPlex NHP Cytokine/Chemokine/GF 37plex (EPX370-40045-901, ThermoFisher Scientific) according to the manufacturer’s instructions.

### RT-PCR

Total RNA from the tissues and cells was extracted and then reverse-transcribed into cDNA using RNeasy Mini Kit (74104, QIAGEN) and PrimeScript RT Master Mix Kit (RR036A, Takara) according to the manufacturers’ instructions. RT-PCR assays were performed using TB Green Premix Ex Taq II (RR820A, Takara) and TaqMan™ Universal Master Mix II (4440039, ThermoFisher Scientific). The sequences of the corresponding primers are listed in Supplementary Table 3.

### Western blot

The samples were lysed and incubated on ice with RIPA lysis buffer (P0013B, Beyotime) supplemented with protease inhibitor (04693159001, Roche) and PMSF (ST505, Beyotime) for 10 min, and then the protein supernatant was collected and quantified with Pierce BCA Protein Assay Kit (23227, ThermoFisher Scientific). Nuclear and Cytoplasmic Protein Extraction Kit (P0028, Beyotime) was employed to extract the nucleoproteins and cytosolic proteins according to the manufacturer’s instructions. After mixed with SDS-PAGE Sample Loading Buffer (P0015L, Beyotime), the protein samples were boiled at 100 °C for 5 min, loaded on SDS-PAGE gels, and transferred to PVDF membranes in sequence. Subsequently, the membranes were blocked with NcmBlot blocking buffer (P30500, NCM Biotech) and incubated with the primary antibodies at 4 °C overnight. The images were obtained after the incubation of secondary antibodies at room temperature for 1 h. The antibodies used in western blot were listed as follows: anti-Tubulin (3873, CST, dilution 1:3000); anti-GAPDH (60004-1-Ig, Proteintech, dilution 1:50000); anti-Lamin B1 (ab16048, Abcam, dilution 1:5000); anti-ACE2 (AF933, Novus, dilution 1:1000); anti-CD147 (MPZ, Jiangsu Pacific Meinuoke Biopharmaceutical Co., Ltd., dilution 1:2000); anti-CD147 (HAb18, our laboratory, dilution 1:3000); anti-CD147 (orb251620, Biorbyt, dilution 1:2000); anti-AHR (67785-1-Ig, Proteintech, dilution 1:5000); anti-AHRR (CSB-PA028950, Cusabio, dilution 1:1000); anti-CYP1A1 (13241-1-AP, Proteintech, dilution 1:1000); anti-BCL2 (60178-1-Ig, Proteintech, dilution 1:1000); anti-Caspase3 (66470-1-Ig, Proteintech, dilution 1:3000); anti-Cleaved caspase3 (9661, CST, dilution 1:1000); anti-MLKL (66675-1-Ig, Proteintech, dilution 1:10000); anti-pMLKL (91689, CST, dilution 1:1000); rabbit anti-goat IgG (H + L) (ZB-2306, ZSGB-BIO, dilution 1: 5000); goat anti-human IgG (A0170, Sigma-Aldrich, dilution 1: 5000); goat anti-mouse IgG (H + L) (31430, ThermoFisher Scientific, dilution 1:5000); goat anti-rabbit IgG (H + L) (31460, ThermoFisher Scientific, dilution 1:5000).

### Multicolor immunofluorescence staining

The slides were dewaxed overnight, followed by antigen retrieval in citrate (pH = 6.0) or Tris-EDTA (pH = 9.0) buffer. After blocked with 5% goat serum, the slides were performed using Opal 6-Plex Manual Detection Kit (NEL811001KT, AKOYA Biosciences) according to the manufacturer’s manuals. The following primary antibodies were used: anti-AGER (16346-1-AP, Proteintech), anti-SFTPC (ab90716, Abcam), anti-CD147 (HAb18, our laboratory), anti-spike RBD (40591-MM43, Sino Biological), anti-CD4 (48274s, CST), anti-CD8 (85336s, CST), anti-CD20 (48750s, CST), anti-CD68 (76437s, CST), anti-Ly6G (ab25377, Abcam), anti-F4/80 (30325s, CST), and anti-iNOS (ab210823, Abcam). Images were analyzed with inForm Tissue Analysis Software (AKOYA Biosciences) and HALO™ Image Analysis Software (Indica labs).

### Hematoxylin & Eosin (H&E) staining and Masson staining

For H&E staining, the tissue sections were deparaffinized by xylene and alcohol, and then counterstained with hematoxylin for 15 min and eosin for 10 min, respectively. Masson staining was performed using Masson Staining Kit (Solarbio, G1340) according to the manufacturer’s manuals.

### SPR assay

The SPR analysis was performed by Biacore T200 (Cytiva). The immobile phases were fixed to CM5 sensor chips (29149603, Cytiva) by amino coupling kit (GE Healthcare, BR-1000-50), including His-CD147 (our laboratory), His-RBD (40592-V08H, Sino Biological), MPZ (Jiangsu Pacific Meinuoke Biopharmaceutical Co., Ltd.), and 3E8 (Wang’s laboratory^38^). The mobile phases contained the mutants of CD147 (His-CD147-R54A, His-CD147-E84A, His-CD147-E92A, His-CD147-Q100A, His-CD147-S112A, our laboratory), the mutants of RBD (His-RBD-G413A, His-RBD-K417A, His-RBD-K424A, His-RBD-G447A, His-RBD-Y489A, Sino Biological), His-RBD (WT) (40592-V08H, Sino Biological), His-RBD (Beta) (40592-V08H85, Sino Biological), His-RBD (Gamma) (40592-V08H86, Sino Biological), His-RBD (JN.1) (40592-V08H155, Sino Biological), rhesus macaque CD147 (Sino Biological), human ACE2 (Sino Biological), and rhesus macaque ACE2 (Sino Biological). The affinity constant for different interacting proteins was analyzed by Biacore T200 Evaluation Software (Cytiva), and the data were illustrated using OriginPro 8.5 (OriginLab).

### Transmission electron microscope (TEM)

The tissues were collected and fixed with PLP Fixative (G2220, Solarbio) for 24 h. After being washed with PBS, the samples were treated with osmic acid for 1.5 h. Subsequently, the tissues were dehydrated with different concentrations of alcohol and soaked in acetone for 15 min. After being embedded and polymerized overnight with epoxy resin, the samples were sliced using ultramicrotome and glued onto copper grid. At last, each section was stained with uranium solution and lead citrate for 10 min, respectively. The ultrastructure was observed by transmission electron microscope (Hitachi).

### Authentic SARS-CoV-2 challenge assay in rhesus macaques

The rhesus macaques (n = 16, male, 3-4 years old) were supplied by Guangxi Grand Forest Scientific Primate Company, which were challenged with 10^6^ TCID_50_ SARS-CoV-2 (Beta strain) by intratracheal routes at 0 dpi and divided into four groups. At 1 dpi, the rhesus macaques were injected intravenously with MPZ or 3E8 antibody. The groups were listed as follows: Ctrl group (PBS), MPZ group (MPZ, 10 mg/kg), 3E8 group (3E8, 10 mg/kg), and MPZ + 3E8 group (MPZ, 5 mg/kg; 3E8, 5 mg/kg). At 3, 5, and 7 dpi, the nasopharyngeal swabs were collected. The animals were euthanized and the tissues were collected at 7 dpi to perform the virus load test, sequencing analysis, and histopathological analysis. The body weight, temperature, CT images, routine blood test, and blood samples were also collected and detected according to the study design, which was shown in Fig. 1a. The tissues of healthy rhesus macaques (n = 4) were kindly supplied by Guangxi Medical University.

### Authentic SARS-CoV-2 challenge assay in hCD147 mice

The hCD147 mice (n = 8, Shanghai Model Organisms) were challenged with 10^5^ TCID_50_ SARS-CoV-2 (Beta strain) intranasally at 0 dpi. The mice were euthanized to collect the tissues for virus load detection and histopathological analysis at 3 dpi (n = 4) and 7 dpi (n = 4). For RBD protein stimulation assay, the hCD147 mice were inoculated with His-RBD protein (n = 4, 20 μg, 40592-V08H, Sino Biological) and 6×His-tag (n = 4, 20 μg, QYAOBIO) intratracheally at -2 dpi. Subsequently, the mice in both groups were challenged with 10^5^ TCID_50_ SARS-CoV-2 (Beta strain) intranasally at 0 dpi and euthanized to collect the tissues at 3 dpi for further analysis. The lung tissues from hCD147 mice inoculated with His-RBD protein (n = 4) and 6×His-tag (n = 4) for 2 days were also collected for the detection of CD147 expression.

### Preparation of single-cell suspensions for scRNA-seq

The scRNA-seq was performed by Genedenovo Biotechnology Co., Ltd. The lung tissues of rhesus macaques (Healthy, n = 4; Ctrl, n = 4; MPZ, n = 4; 3E8, n = 4; MPZ + 3E8, n = 4; at 7dpi) and virus-infected hCD147 mice (n = 4; at 3dpi) were cut into 1–2 mm^3^ pieces in DPBS buffer and digested with collagenase I and DNase I at 37 °C for 30–60 min with persistent shaking every 10 min. The digested tissues were then passed through a 70 μm mesh filter, and the cell suspension was centrifuged at 4 °C for 5 min (350 g). The cell pellet was suspended in red blood cell removal solution at 4 °C for 10 min and then resuspended in DPBS buffer (0.04% BSA and 0.01 mM EDTA) after being washed by DPBS buffer. Following the depletion of the dead cells, the cell suspensions were directly performed for scRNA-seq according to the manufacturer’s instructions.

### ScRNA library preparation and sequencing

For the samples from rhesus macaques, the cells in single-cell suspension were counted by trypan blue dye at a proper concentration of 1000–2000 cells/μL. Single-cell suspensions were loaded onto 10× Genomics Chromium v3.1 system to generate single-cell Gel Beads-In-Emulsions (GEMs), where all generated full-length cDNA share a common 10× barcode. Then, GEMs were disrupted and cDNA was amplified via PCR. The quality control of the sequencing libraries was performed by High Sensitivity DNA assay Kit (5067-4626, Agilent Technologies), and the concentration quantification was conducted using ABI StepOnePlus Real-Time PCR System. Finally, the sequencing was performed on Illumina sequencing platform in PE150 mode. For the samples from hCD147 mice, cell suspensions were barcoded through the SeekOne single cell platform.

### Sample and library preparation for spatial transcriptome sequencing

The spatial transcriptome sequencing was performed by Genedenovo Biotechnology Co., Ltd. The lung tissues of rhesus macaques (Healthy, n = 4; Ctrl, n = 4; MPZ, n = 4; 3E8, n = 4; MPZ + 3E8, n = 4; at 7dpi) and virus-infected hCD147 mice (n = 4; at 3dpi) were used to perform spatial transcriptome sequencing. The tissues were embedded in the OCT-filled mold and cut into slices by cryo-sectional technology. Subsequently, the samples were barcoded through 10× Genomics Platform using Visium Spatial Tissue Optimization Reagent Kit and Slide Kit (PN-1000192, PN-1000191, 10× Genomics), Visium Spatial Gene Expression Reagent Kit and Slide Kit (PN-1000189, PN-1000188, 10× Genomics), Library Construction Kit (PN-1000196, 10× Genomics), and Dual Index Kit (PN-1000215, 10× Genomics). Each sequencing library was sequenced using Illumina sequencing platform.

### The quality control and processing for scRNA-seq data of rhesus macaques

Cellranger (v7.1.0) was used to process scRNA-seq data with macaque reference Ensembl_release105. Seurat (v4.1.0) was used for processing expression matrices. The cells with less than 200 and more than 5000 genes were filtered. Cells with less than 25000 nUMI detected and less than 10 percent of detected reads coming from mitochondrial genes were finally retained. DoubletFinder (v2.0.3) and harmony (v0.1.0) was set to default parameter.^73^

### Quality control for scRNA-seq data of hCD147 mice

ScRNA-seq data was aligned and quantified using Seeksoultools (v1.2.0) and STAR (v2.7.1a) against the Ensembl_release111 added with SARS-CoV-2 Beta reference genome. The mouse CD147 sequence in the reference genome was replaced with human CD147 sequence. Raw counts were then used for downstream analysis. Seurat (v4.1.0) was used to process the gene expression matrix. Cells with SARS-CoV-2 genes detected were labeled as infected cells. Cells with less than 200 and more than 5600 genes were filtered. Cells with less than 12000 nUMI detected and less than 33 percent of detected reads coming from mitochondrial genes were finally kept. To remove the deviation of double cells, DoubletFinder (v2.0.3) was used with pN parameter of 0.25 and nExp parameter of 4 × 10^-6^. The remaining cells were then used for gene expression matrix logarithmic normalization and scaling. To integrate cells into a shared space from different datasets for unsupervised clustering, harmony (v0.1.0) was used to correct the batch effect by default parameters.

### ScRNA-seq data analyses

Principal components were initially derived to diminish the dimensionality of the feature space before conducting clustering. The first 50 principal components and batch-corrected matrix were selected for nearest neighbor graph building and dimensionality reduction clustering. Clustering resolution parameter was set to 0.5. Uniform manifold approximation and projection (UMAP) was used to illustrate the similarities among different clusters. Differentially expressed genes among clusters were determined using Wilcoxon rank-sum tests, only considering genes that demonstrated a log_2_(fold change) exceeding 0.25 between clusters, *p* value less than 0.05 and log_2_(fold change) more than 0.36. Genes with the highest log_2_(fold change) and classical cell type marker genes were applied in cell annotation. Details of the data analyses were provided in the Supplementary Materials.

### Spatial transcriptome sequencing data analyses in rhesus macaques

Spaceranegr (v1.2.1) was used to process spatial sequencing data for mapping to reference genome. SCTransform function in Seurat (v4.1.0) was used to process the gene expression matrix. The pathologists preliminarily divided the pathological areas of the sections in virus-infected group, integrated the matrix and gene expression matrix after stlearn sorted out the H&E sections, and then performed dimensionality reduction clustering, which was defined according to the data of pathological spots in each cluster.^74^ The Normal and Lesion regions are defined according to the following formula:

$${\rm{C}}\left({{\rm{n}}}_{{\rm{A}}},\,{{\rm{n}}}_{{\rm{B}}}\right)=\left\{\begin{array}{l}{\rm{A}}\,\mathrm{if}\,\left({{\rm{n}}}_{{\rm{A}}}\, > \,5\,\mathrm{and}\,{{\rm{n}}}_{{\rm{A}}}\ge 3{{\rm{n}}}_{{\rm{B}}}\right)\\ {\rm{B}}\,\mathrm{if}\,\left({{\rm{n}}}_{{\rm{B}}}\, > \,5\,\mathrm{and}\,{{\rm{n}}}_{{\rm{B}}}\ge 3{{\rm{n}}}_{{\rm{A}}}\right)\\ \mathrm{undefine}\end{array}\right.$$

runRCTD function in spacexr (v2.2.1) was used for assigning single cell types to spatial transcriptomics spots and defining a spot where the proportion of a certain cell type exceeds 50% as that cell type.^75^

### Spatial transcriptome sequencing data analyses in hCD147 mice

Spaceranegr (v1.3.1) was used to process mouse spatial sequencing data for mapping to reference genome. SCTransform function in Seurat (v4.1.0) was used to process the gene expression matrix. Spots with SARS-CoV-2 genes was defined as infected spots; otherwise, defined as uninfected spots. On this basis, the two uninfected positions closest to the infected positions are defined as border positions. runRCTD function in spacexr (v2.2.1) was used for assigning single cell types to spatial transcriptomics spots and defining a spot where the proportion of a certain cell type exceeds 50% as that cell type.

### CellChat analysis

The data of AT1, AT2, B cells, CD4 + T cells, CD8 + T cells, monocytes, and macrophages was extracted from the healthy, infected, and antibody treatment groups of rhesus macaque model to construct CellChat objects. To assess differences in cellular communication across any two groups, we employed the mergeCellChat function to create a comparison object, and subsequently used the netVisual_diffInteraction function to quantify and compare the inter-group variations in cellular communication. In the presentation of the results, red lines denote an increase in cellular communication in infected group or antibody treatment group compared to the healthy group or infected group, while blue lines denote a decrease. The thickness of the lines represents the communication strength. Subsequently, we classified macrophages into subgroups, reconstructed the CellChat object for each subgroup, extracted the *p*-values and probabilities of ligand-receptor pairs for each subgroup, and plotted the significant ligand-receptor pairs as a heatmap. The above analytical method is also applied to the CellChat analysis for the data from virus-infected hCD147 mice.

### Statistical analysis

Data were analyzed with GraphPad Prism Software. Significant differences were analyzed by unpaired Student’s t test, unpaired t test with Welch’s correction, or two-way ANOVA. Data are displayed as dot plots of at least three independent experiments (mean ± SEM). For single cell sequencing data, the Wilcoxon rank-sum tests were used to detect differential genes between the two groups. All statistical analyses were performed using R (v.4.1.3). The statistical significance was determined as *p < 0.05, **p < 0.01, and ***p < 0.001.

## Supplementary information

Supplementary Materials

## Data Availability

Cryo-EM density maps for the CD147-SARS-CoV-2 spike trimer complex have been deposited in the Electron Microscopy Data Bank (EMDB) under accession code EMD-64131. The corresponding atomic models have been deposited in the Protein Data Bank (PDB) with accession code 9UG3. The original data of scRNA-seq and spatial transcriptomics has been uploaded to the GEO database (GSE298097 and GSE298098).
