# Supplementary material for: Inducible CD147 up-regulation boosts extended SARS-CoV-2 infection triggering severe COVID-19 independent of ACE2
Source: Signal Transduct Target Ther. 2026 Feb 3;11:42. doi: 10.1038/s41392-025-02551-x (PMC12864993; doi:10.1038/s41392-025-02551-x)
Supplement: Supplementary file 1 — Supplementary Materials [file 41392_2025_2551_MOESM1_ESM.docx]

Supplementary Materials for

**Inducible CD147 up-regulation boosts extended SARS-CoV-2 infection triggering severe COVID-19 independent of ACE2**

Ke Wang^1,2#^, Peng Lin^1,2#^, Ruo Chen^1,2#^, Qiang Huang^2,3#^, Yizhen Zhao^4#^, Lei Zhang^4^, Yongxiang Zhao^5^, Liping Zhong^5^, Ke Xu^6^, Linlin Bao^7^, Youchun Wang^8^, Chuan Qin^7^, Guizhen Wu^6^, Hai Zhang^1,2^, Jiejie Geng^1,2^, Zheng Zhang^1,2^, Ding Wei^1,2^, Xiaochun Chen^9^, Hao Tang^9^, Liu Yang^1,2^, Xu Yang^1,2^, Xiuxuan Sun^1,2^, Rui Yao^1,2^, Ye Zhao^1,2^, Weijun Qin^10*^, Zhiwei Yang^4*^, Liang Chen^2,3*^, Huijie Bian^1,2*^, Zhi-Nan Chen^1,2*^, and Ping Zhu^1,2*^

Correspondence to: qinwj@fmmu.edu.cn, yzws-123@xjtu.edu.cn, lchen1@shu.edu.cn, hjbian@fmmu.edu.cn, znchen@fmmu.edu.cn, zhuping@fmmu.edu.cn

**This PDF file includes:**

Figures S1 to S10

Tables S1 to S3


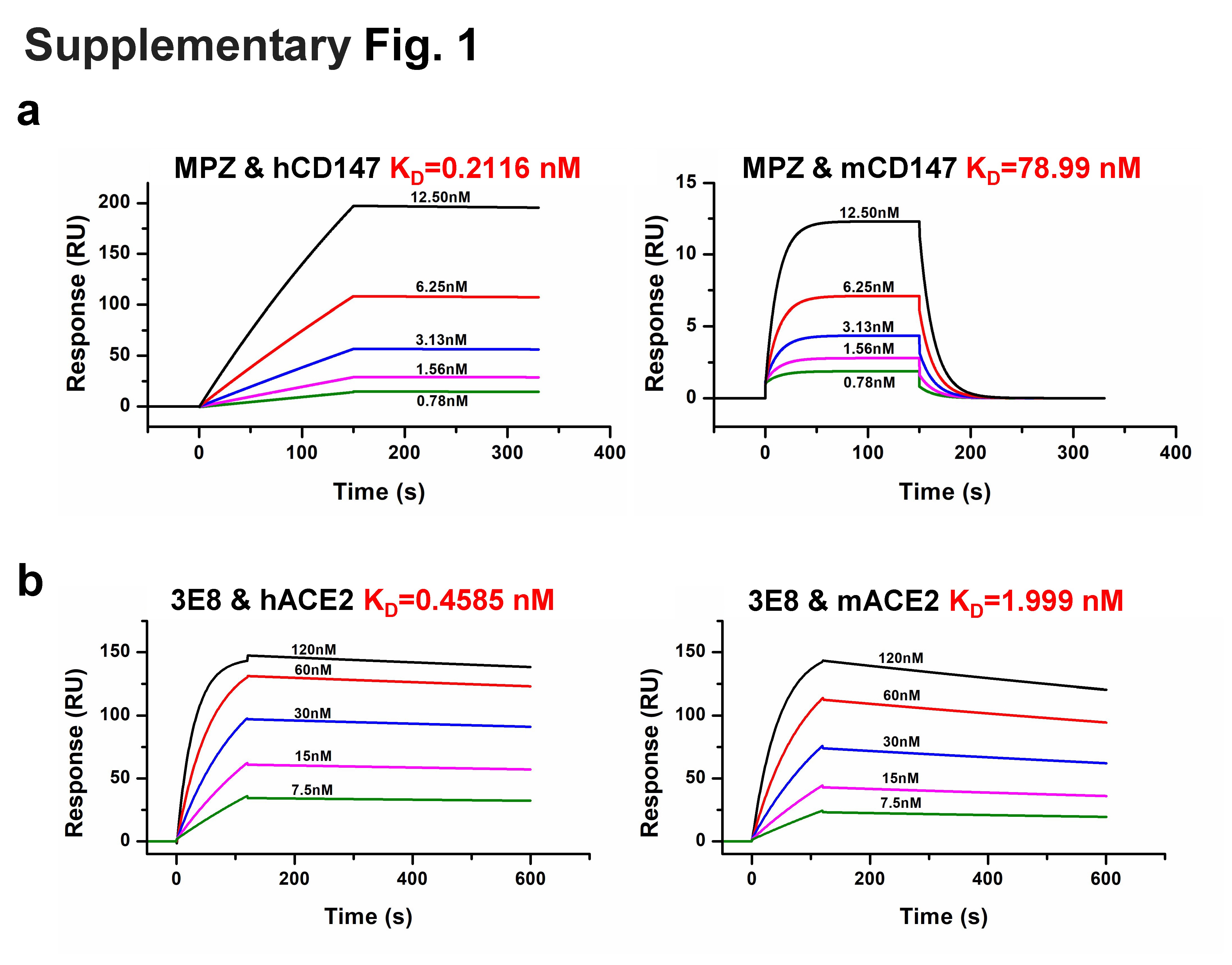


Supplementary Fig. 1. SPR assays show the binding ability of MPZ or 3E8 to CD147 and ACE2. a, The binding ability of MPZ and human CD147 (hCD147) or rhesus macaque CD147 (mCD147) was determined by SPR assays, MPZ & hCD147, K_D_=0.2116 nM, MPZ & mCD147, K_D_=78.99 nM. b, The binding ability of 3E8 and human ACE2 (hACE2) or rhesus macaque ACE2 (mACE2) was determined by SPR assays, 3E8 & hACE2, K_D_=0.4585 nM, 3E8 & mACE2, K_D_=1.999 nM.


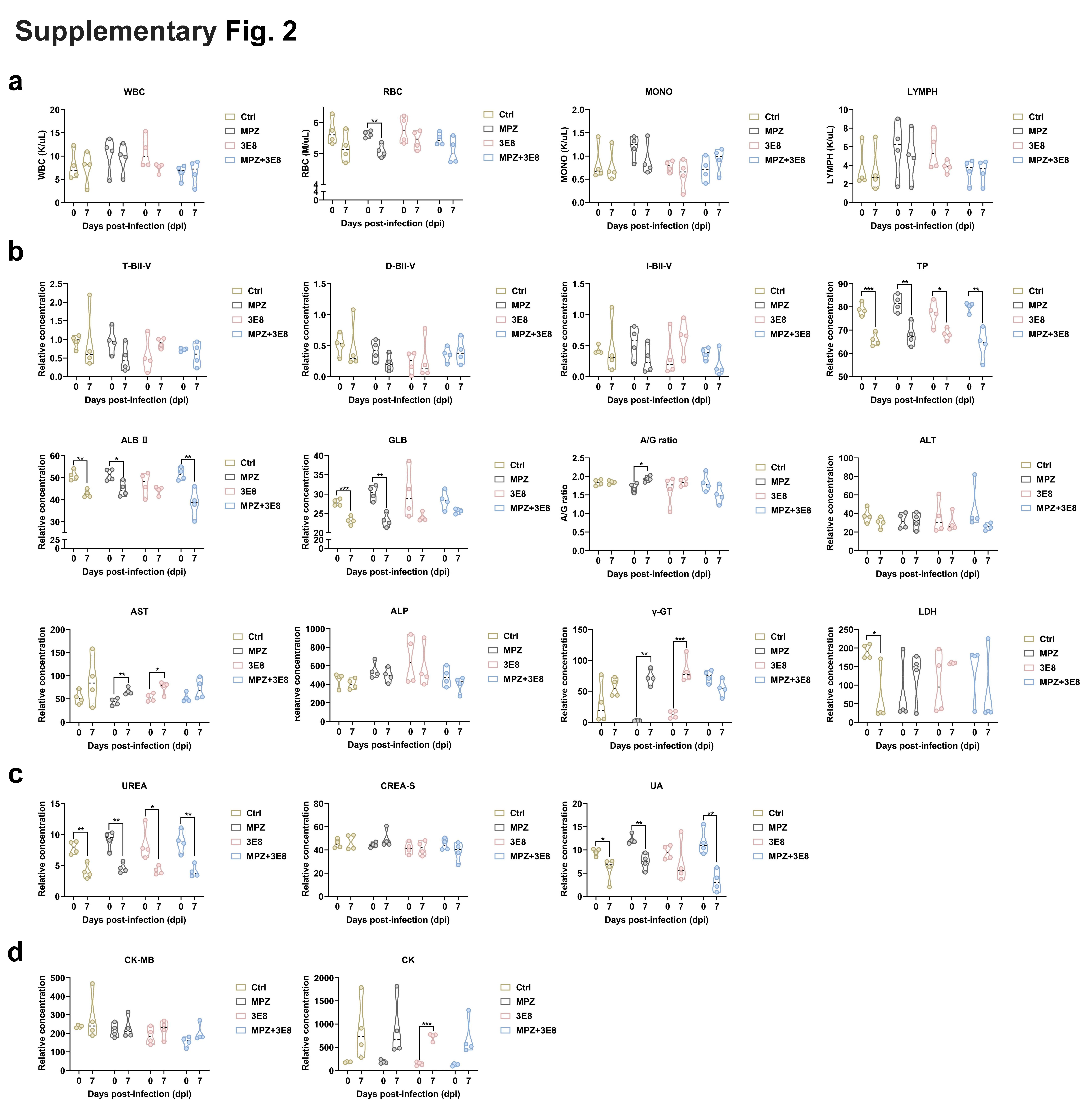


Supplementary Fig. 2. The detection of blood routine and serum biochemical parameters in COVID-19 model of rhesus macaques. a, The blood routine test was performed in COVID-19 model of rhesus macaques at 0 and 7 dpi. b-d, The serum biochemical parameters were tested in COVID-19 model of rhesus macaques at 0 and 7 dpi, including liver function test (b), kidney function test (c), and myocardial enzyme detection (d).





Supplementary Fig. 3. The pathological characteristics of lung tissues in COVID-19 model of rhesus macaques. a-c, The pathological features of lung tissues from rhesus macaques were performed by H&E staining, scale bar, 500 μm (a) and electron microscope, scale bar, 20 μm (b,c). The green arrows indicate the pathological features of the lung tissues. d, Electron microscope shows virus-like particles in AT2 cell, endothelial cell, fibroblast, lymphocyte, macrophage, and neutrophil of virus-infected lung tissues from rhesus macaques. The virus-like particles were indicated with red arrows, scale bar, 1 μm. e,f, The collagen deposition in the lung tissues of virus-infected rhesus macaques was evaluated using masson staining, scale bar, 2000 μm (e), and the relative percentage of positive area in lung tissues was analyzed in all groups, **p*<0.05, ***p*<0.01 (f).


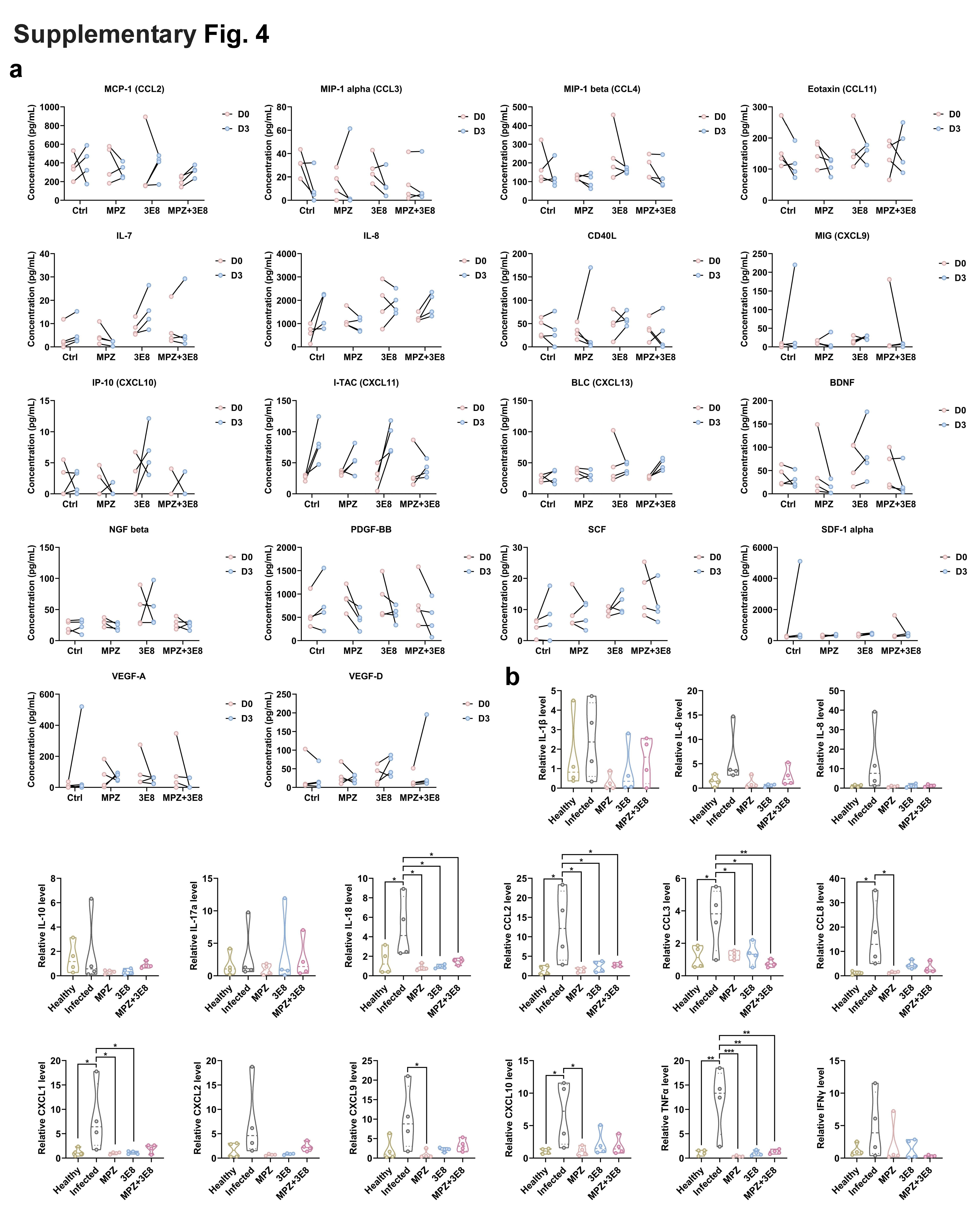


Supplementary Fig. 4. The detection of cytokines, chemokines, and growth factors in serum or lung tissues of virus-infected rhesus macaques. a, The cytokines, chemokines, and growth factors in serum of rhesus macaques were evaluated by multifactor kit at 0 and 3 dpi. b, The level of cytokines and chemokines in lung tissues of virus-infected rhesus macaques were determined by RT-PCR, **p*<0.05, ***p*<0.01, ****p*<0.001.


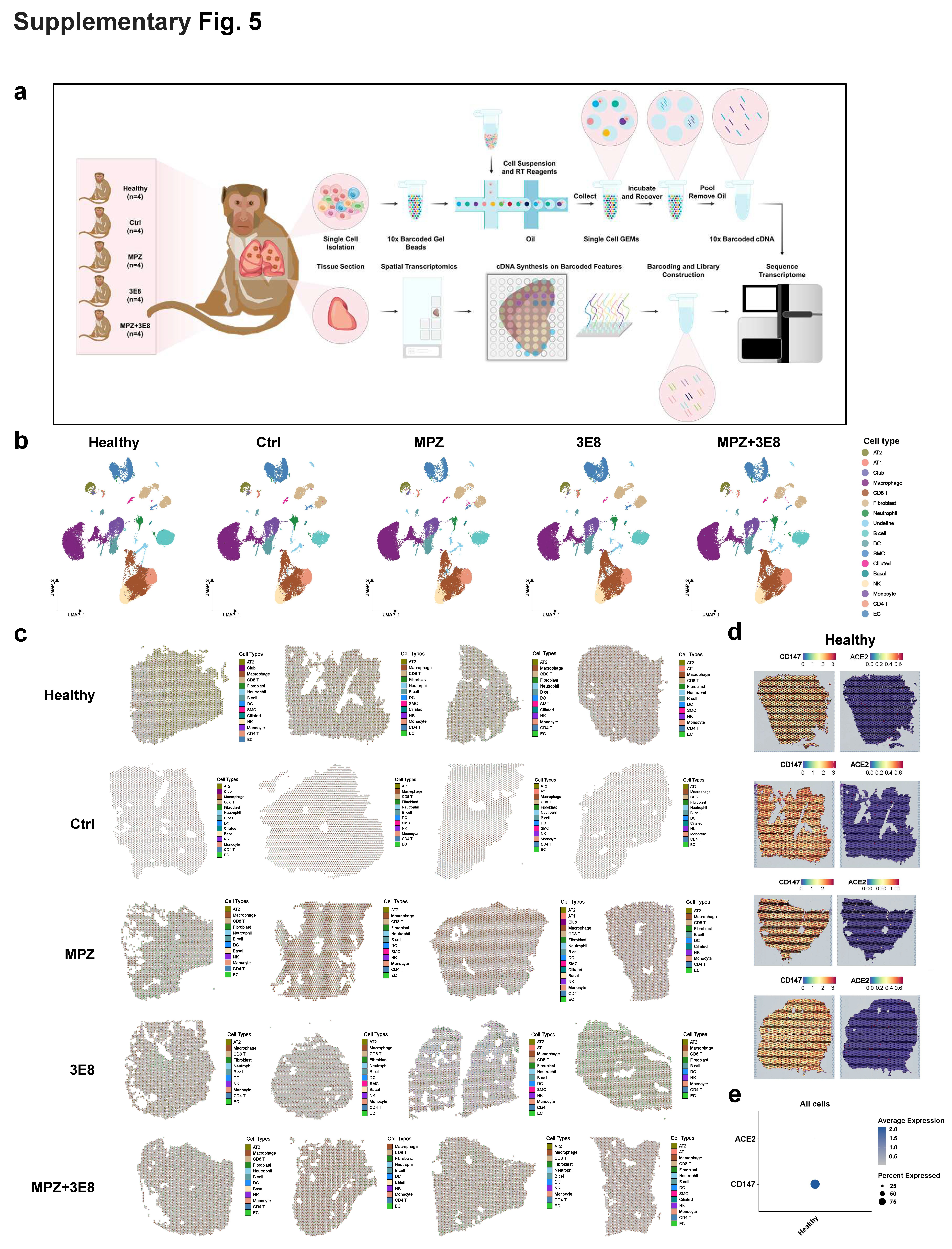


Supplementary Fig. 5. The scRNA-seq and spatial transcriptomic analysis of lung tissues in rhesus macaque model of COVID-19. a, The experimental schematic for scRNA-seq and spatial transcriptomic analysis using lung tissues of rhesus macaque model of COVID-19, including healthy (n=4), Ctrl (n=4), MPZ (n=4), 3E8 (n=4), and MPZ+3E8 (n=4). b, The UMAP of scRNA-seq was presented with seventeen cell types in healthy, Ctrl, MPZ, 3E8, and MPZ+3E8 groups. c, The cell types of scRNA-seq data were mapped to spatial transcriptomic data of rhesus macaques. d,e, The spatial transcriptomic analysis identified the expression of CD147 and ACE2 in the lung tissues of healthy rhesus macaques (d), and their expressions were quantified (e).


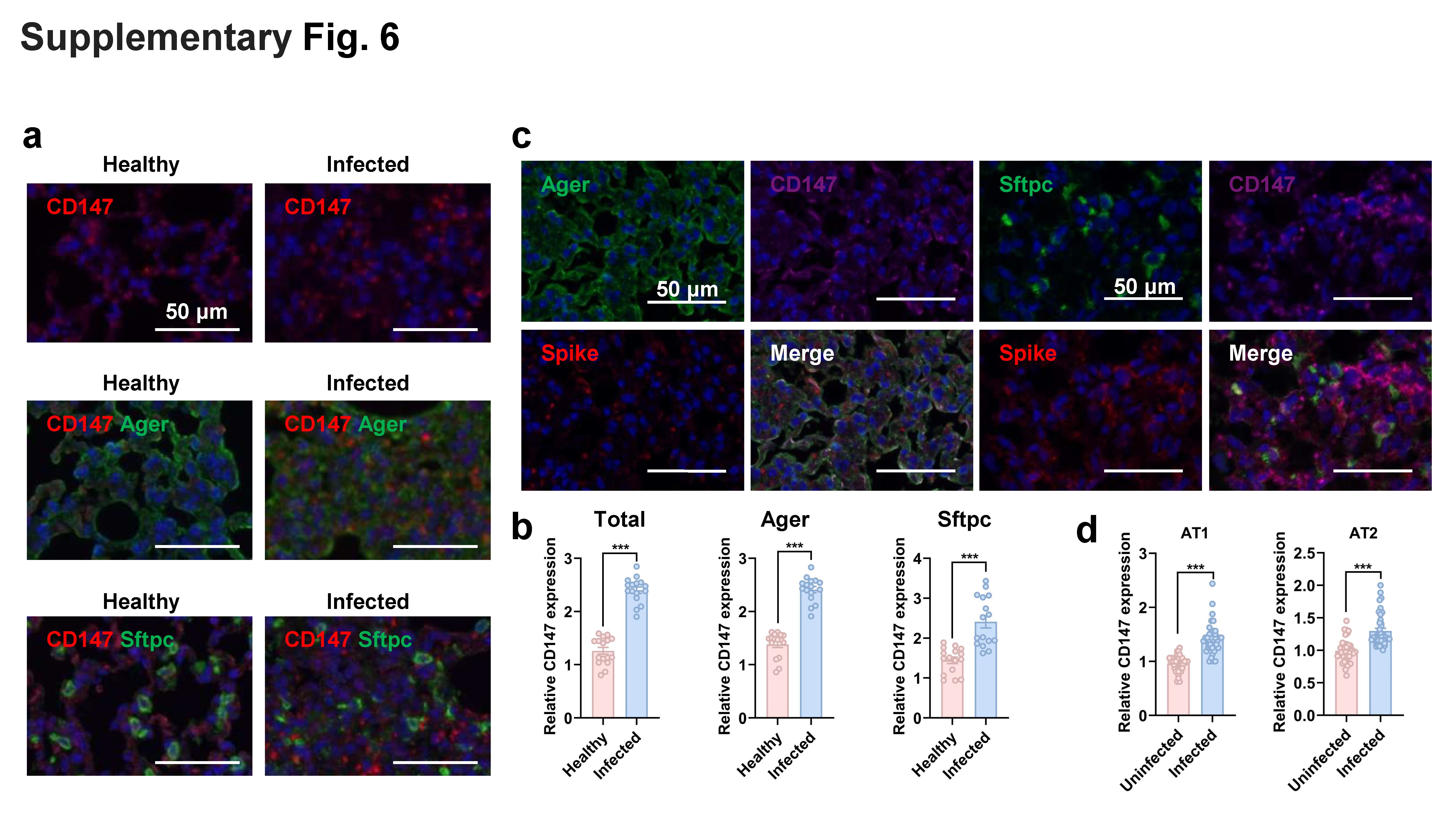


Supplementary Fig. 6. SARS-CoV-2 infection induces CD147 up-regulation in the lung tissues of hCD147 mice. a,b, The expression of CD147 (red) was detected in the lung tissues of healthy and virus-infected hCD147 mice using multicolor immunofluorescence staining, and ager (green) and sftpc (green) were used to indicate AT1 and AT2 cells, respectively, scale bar, 50 μm (a). The quantities of CD147 were analyzed using inForm Tissue Analysis Software and HALO™ Image Analysis Software, ****p*<0.001 (b). c,d, The expression of CD147 (purple) was performed in the lung tissues of virus-infected hCD147 mice using multicolor immunofluorescence staining, and spike (red), ager (green), and sftpc (green) were used to indicate virus-infected cells, AT1, and AT2 cells, respectively, scale bar, 50 μm (c). The quantities of CD147 in AT1 and AT2 cells with virus infection were analyzed using inForm Tissue Analysis Software and HALO™ Image Analysis Software, ****p*<0.001 (d).


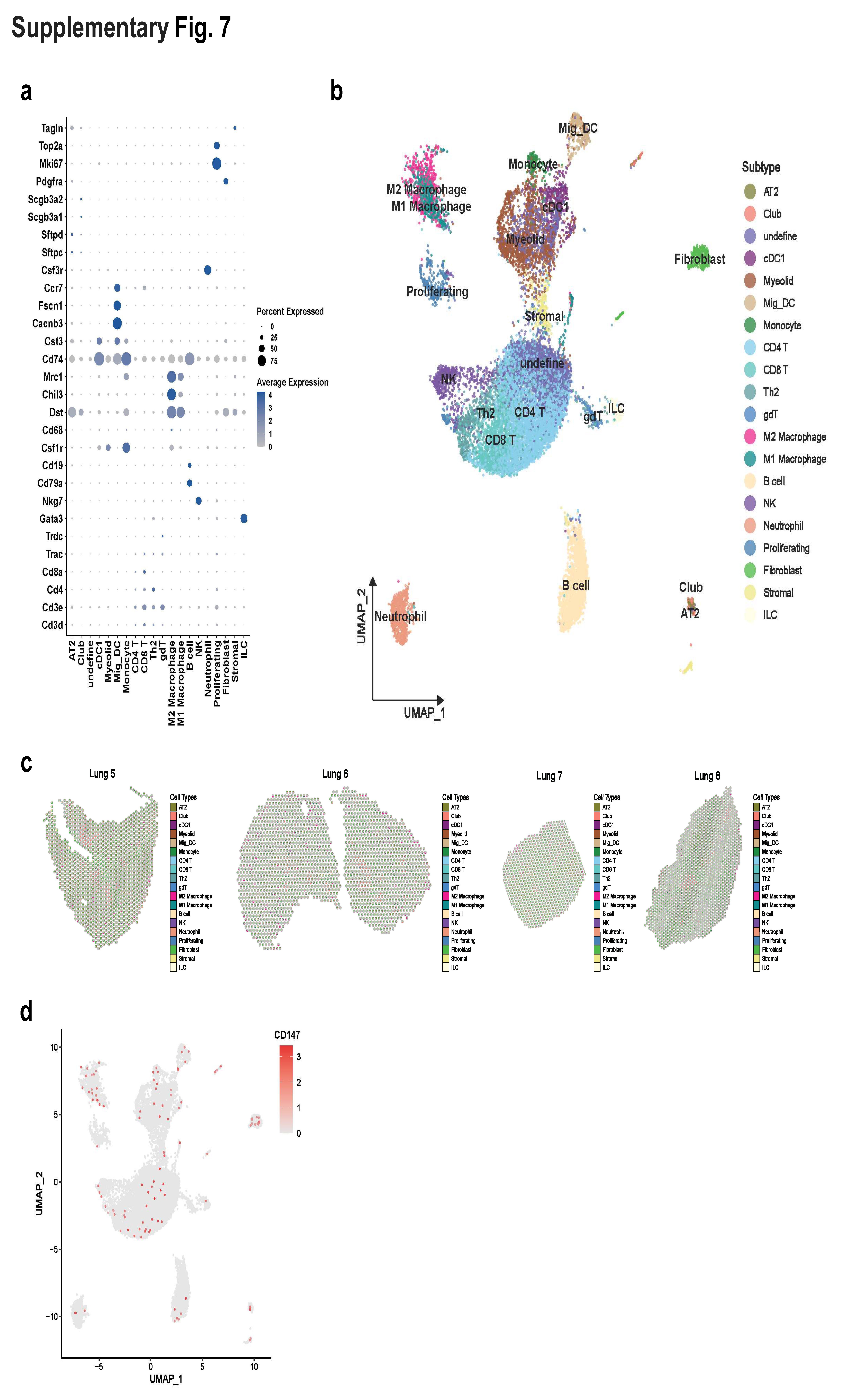


Supplementary Fig. 7. The scRNA-seq and spatial transcriptomic analysis of lung tissues in hCD147 mouse model of COVID-19. a,b, The clusters of scRNA-seq were identified by marker genes of corresponding cell types (a), and the UMAP was presented with twenty cell types (b). c, The cell types of scRNA-seq data were mapped to spatial transcriptomic data of hCD147 mice. d, The expression of human CD147 was displayed in different cell types of lung tissues in hCD147 mouse model of COVID-19.





Supplementary Fig. 8. The scRNA-seq analysis of lung tissues in rhesus macaque model of COVID-19 and the detection of pseudovirus infection in immune cells. a, Data from scRNA-seq showed the percentages of different cell types in the lung tissues of rhesus macaques in five groups, **p*<0.05. b,c, ScRNA-seq data of the lung tissues in healthy and virus-infected rhesus macaques showed the expression levels of cell death markers in CD4+ T cells (b) and B cells (c). d, The expressions of CD147 and ACE2 in CD4+ T cells and B cells were determined by western blot. e, The relative luciferase signals were determined by dual-luciferase reporter assays in pseudovirus-infected CD4+ T cells and B cells, ****p*<0.001. f,g, The macrophages in scRNA-seq of rhesus macaques were re-clustered into three subtypes, namely M0, M1, and M2 macrophages (f), and the UMAP was presented (g). h, Data from scRNA-seq showed the percentage of M0, M1, and M2 macrophages in the lung tissues of rhesus macaques in five groups.


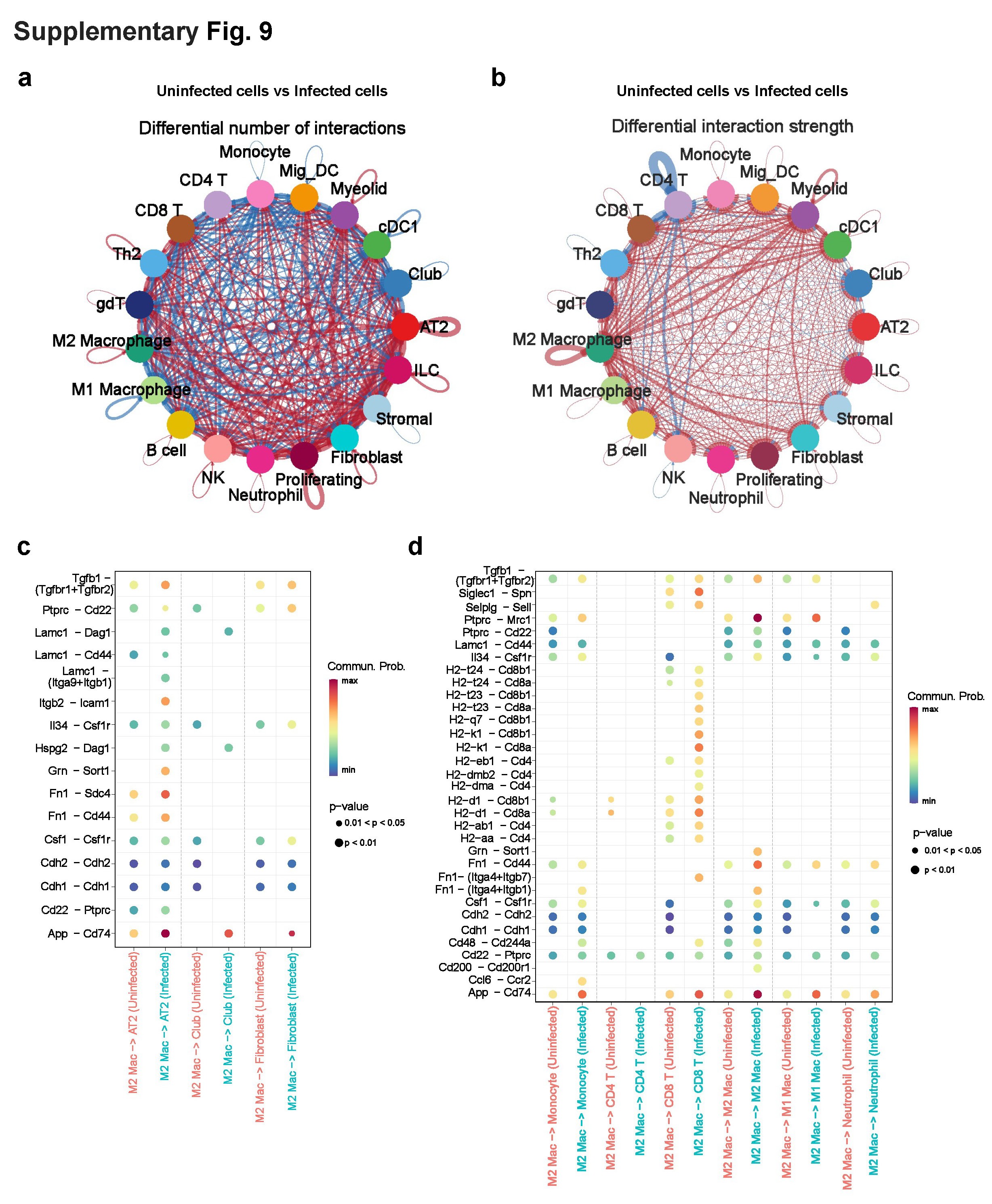


Supplementary Fig. 9. SARS-CoV-2 infection elicits abnormal cell-cell communications among different cell types in the lung tissues of hCD147 mice. a,b, ScRNA seq analysis showed abnormal cell-cell communications among different cell types in the lung tissues of virus-infected hCD147 mice, including interaction number (a) and interaction strength (b). c,d, ScRNA seq analysis demonstrated the cell-cell communications in the lung tissues of virus-infected hCD147 mice between M2 macrophages and other cell types, including non-immune cells (c) and immune cells (d).





Supplementary Fig. 10. Cryo-EM data collection and structure determination of CD147-spike complex. a, Representative micrograph (magnification 96,000×, defocus -1.6 µm) of the CD147-spike complex specimen, scale bar, 50 nm. b, Representative 2D class averages of the unliganded spike and CD147-spike complex. c, Representative 2D class averages of the CD147-spike complex, with CD147-ECD clearly visible in some 2D class averages (green arrows). d, Flow chart of cryo-EM data processing of the CD147-spike complex, including particle selection, classifications, and density map reconstruction. e, The Fourier shell correction (FSC) between two independently reconstructed volumes, indicating the resolution of the 3D reconstruction using the FSC=0.143 criterion. f, Backbone-atom RMSD of the CD147-spike complex with respect to simulation time (100 ns). g, The structure of CD147-spike complex was shown as cartoons fitted into the cryo-EM map surface. The cryo-EM map was displayed as a semi-transparent surface. h, The side and top views of the overlaid CD147-spike (colored) and spike-closed (dark gray) structures, showing a 32.3° upward/outward rotation of the RBD in the up protomer during the spike binding to CD147. i, The rotations of the NTD from the spike-closed (gray) to the CD147-spike (colored) state, with the NTD also exhibiting a downward/outward movement. j, Surface representation of the spike RBD (green) highlighting the interfaces involved in CD147 (left, yellow) and ACE2 (right, pink) recognition, with both images presented from the same viewpoint. The structure of ACE2 complexed with the spike RBD of SARS-CoV-2 was retrieved from the Protein Data Bank (PDB ID: 6M0J).

Supplementary Table 1. The binding free energies (*ΔGbind*)

| **Complexes** | ***ΔE_ele_*** | ***ΔE_vdw_*** | ***ΔE_surf_*** | ***ΔE_GB_*** | ***ΔG_bind_*** |
| --- | --- | --- | --- | --- | --- |
| **CD147-spike** | -514.53 ± 3.09 | -182.16 ± 0.83 | -23.88 ± 0.05 | 572.35 ± 2.62 | -148.22 ± 0.90 |
| **CD147-spike (Beta)**  **CD147-spike (Gamma)**  **CD147-spike (JN.1)** | -504.52 ± 3.25  -441.71 ± 3.76  -1222.54 ± 5.72 | -186.41 ± 0.67  -184.18 ± 0.89  -174.12 ± 0.98 | -24.83 ± 0.07  -23.75 ± 0.07  -25.37 ± 0.07 | 565.08 ± 2.95  512.31 ± 3.52  1234.07 ± 5.19 | -150.67 ± 0.86  -137.33 ± 0.97  -160.41 ± 1.02 |

All values are given in *kcal/mol*$\mathrm{kcal}\mathrm{mol}^{-1}$, and the values behind “±” are their standard deviations (SD).

Supplementary Table 2. Statistics of the structural models of SARS-CoV-2 spike-CD147 complex at 3.75 Angstroms resolution

| **Model statistics** | **SARS-CoV-2 spike-CD147** |
| --- | --- |
| **PDB** | 9UG3 |
| **R.m.s. deviations** |  |
| Bond lengths (Å) | 0.021 |
| Bond angles (°) | 2.193 |
| **Validation** |  |
| MolProbity score | 1.92 |
| Clash score | 3.11 |
| **Ramachandran plot (%)** |  |
| Favored | 88.40 |
| Allowed | 11.15 |
| Outliers | 0.45 |
| **Rama-Z** |  |
| Whole | -3.09 (0.12) |
| Helix | -1.87 (0.16) |
| Sheet | -0.80 (0.25) |
| Loop | -2.51 (0.11) |
| **Model vs. Data** |  |
| CC (mask) | 0.62 |
| CC (box) | 0.76 |
| CC (volume) | 0.60 |
| CC (peaks) | 0.60 |

Supplementary Table 3. The sequences of the corresponding primers

| **Primer name** | **Forward (5'-3')** | **Reverse (5'-3')** |
| --- | --- | --- |
| Actin (mouse) | GTGACGTTGACATCCGTAAAGA | GCCGGACTCATCGTACTCC |
| TNF-α (mouse) | CCCTCACACTCAGATCATCTTCT | GCTACGACGTGGGCTACAG |
| IL-1β(mouse) | CAGCACATCAACAAGAGCTTCAG | GAGGATGGGCTCTTCTTCAAAGA |
| IL-6 (mouse) | TGGAGTACCATAGCTACCTGGA | TGGAAATTGGGGTAGGAAGGAC |
| IL-8 (mouse) | TCGAGACCATTTACTGCAACAG | CATTGCCGGTGGAAATTCCTT |
| IL-17a (mouse) | TCAGCGTGTCCAAACACTGAG | CGCCAAGGGAGTTAAAGACTT |
| IL-18 (mouse) | GTCCTGCCTCAAACAAACATGTA | AGAAAGCATGGAACCACAGAGAA |
| CCL2 (mouse) | TCACCAGCAAGATGATCCCA | CTTGAGCTTGGTGACAAAAACTA |
| CCL3 (mouse) | TTCTCTGTACCATGACACTCTGC | CGTGGAATCTTCCGGCTGTAG |
| CCL8 (mouse) | CTGGGCCAGATAAGGCTCC | CATGGGGCACTGGATATTGTT |
| CXCL1 (mouse) | GGTCTGAGTCCTCGCTCAAG | GTCGCACCTCCACATAGCTT |
| CXCL2 (mouse) | CCCAGACAGAAGTCATAGCCAC | TGGTTCTTCCGTTGAGGGAC |
| CXCL9 (mouse) | TCCTTTTGGGCATCATCTTCC | TTTGTAGTGGATCGTGCCTCG |
| CXCL10 (mouse) | GGTCTGAGTCCTCGCTCAAG | GTCGCACCTCCACATAGCTT |
| GAPDH (human/monkey) | GCACCGTCAAGGCTGAGAAC | TGGTGAAGACGCCAGTGGA |
| TNFα (monkey) | GAGCACTGAAAGCATGATCCG | GGGGTCCTTGGGGAACTCTTC |
| IFN-γ (monkey) | AGGAATTGGAAAGAGGAGAGTGA | AGGAATTGGAAAGAGGAGAGTGA |
| IL-1β (human/monkey) | ATGATGGCTTATTACAGTGGCAA | GTCGGAGATTCGTAGCTGGA |
| IL-6 (monkey) | TCTGGATTCAATGAGGACACTT | GTTGGTTCAGGGGTGGTTATT |
| IL-8 (monkey) | CGGAAGGAACCATCTCGCTC | GGCAAAACTGCACCTTCACAC |
| IL-10 (monkey) | CTGAGAACCACGACCCAGAC | CGCCTTTCTCTTGGAGCTTAC |
| IL-17a (human/monkey) | TCCCACGAAATCCAGGATGC | GGATGTTCAGGTTGACCATCAC |
| IL-18 (monkey) | ACAGTACGCTTTACTTTATAGCTGA | ATGGTCCGGGGTGCATTATC |
| CCL2 (monkey) | CCCAAAGAAGCTGTGATCTTCAA | TCAAGGCTTCGGAGTTTGGAT |
| CCL3 (monkey) | CAAGCCCGGTGTCATCTTCC | CAAGCCCGGTGTCATCTTCC |
| CCL8 (monkey) | GACTTGCTCAGCCAGATTCAG | TCACAGCTTCCTTGGGACAC |
| CXCL1 (monkey) | CCGAAACCGAAGTCATAGCC | TTGGATTTGTCACAGTTCAGC |
| CXCL2 (monkey) | CCAAACCGAAGTCATAGCCAC | AGGAACAGCCACCAATGAGC |
| CXCL9 (monkey) | ATTGGAGTGCAAGGAACCCC | TCTCGCAGGAAAGGTTTGGA |
| CXCL10 (monkey) | CCACATGTTGAGATCATTGCTAC | ACCTTTCCTTGCTAACTGCTT |
| SARS-CoV-2 N | GGGGAACTTCTCCTGCTAGAAT | CAGACATTTTGCTCTCAAGCTG |
